# CellSIUS provides sensitive and specific detection of rare cell populations from complex single-cell RNA-seq data

**DOI:** 10.1186/s13059-019-1739-7

**Published:** 2019-07-17

**Authors:** Rebekka Wegmann, Marilisa Neri, Sven Schuierer, Bilada Bilican, Huyen Hartkopf, Florian Nigsch, Felipa Mapa, Annick Waldt, Rachel Cuttat, Max R. Salick, Joe Raymond, Ajamete Kaykas, Guglielmo Roma, Caroline Gubser Keller

**Affiliations:** 1 Novartis Institutes for Biomedical Research, Basel, Switzerland; 2 Novartis Institutes for Biomedical Research, Cambridge, USA; 3 Present Address: Institute of Molecular Systems Biology, ETH Zurich, Zurich, Switzerland; 4 Present Address: Insitro, San Francisco, USA; 5 Present Address: Flagship Pioneering, Cambridge, USA

**Keywords:** Single-cell RNA sequencing, Data analysis, Rare cell types, Clustering, Software, Benchmarking, Human pluripotent stem cells, Cortical development, Choroid plexus, Lineage mapping

## Abstract

We develop CellSIUS (Cell Subtype Identification from Upregulated gene Sets) to fill a methodology gap for rare cell population identification for scRNA-seq data. CellSIUS outperforms existing algorithms for specificity and selectivity for rare cell types and their transcriptomic signature identification in synthetic and complex biological data. Characterization of a human pluripotent cell differentiation protocol recapitulating deep-layer corticogenesis using CellSIUS reveals unrecognized complexity in human stem cell-derived cellular populations. CellSIUS enables identification of novel rare cell populations and their signature genes providing the means to study those populations in vitro in light of their role in health and disease.

**Electronic supplementary material:**

The online version of this article (10.1186/s13059-019-1739-7) contains supplementary material, which is available to authorized users.

## Background

Single-cell RNA sequencing (scRNA-seq) enables genome-wide mRNA expression profiling with single-cell granularity. With recent technological advances [1, 2] and the rise of fully commercialized systems [3], throughput and availability of this technology are increasing at a fast pace [4]. Evolving from the first scRNA-seq dataset measuring gene expression from a single mouse blastomere in 2009 [5], scRNA-seq datasets now typically include expression profiles of thousands [1–3] to more than one million cells [6, 7]. One of the main applications of scRNA-seq is uncovering and characterizing novel and/or rare cell types from complex tissue in health and disease [8–13].

From an analytical point of view, the high dimensionality and complexity of scRNA-seq data pose significant challenges. Following the platform development, a multitude of computational approaches for the analysis of scRNA-seq data emerged. These comprise tools for cell-centric analyses, such as unsupervised clustering for cell type identification [14–16], analysis of developmental trajectories [17, 18], or identification of rare cell populations [8, 9, 19], as well as approaches for gene-centric analyses such as differential expression (DE) analysis [20–22].

Whereas a large number of computational methods tailored to scRNA-seq analysis are available, comprehensive performance comparisons between those are scarce. This is mainly due to the lack of reference datasets with known cellular composition. Prior knowledge or synthetic data are commonly used to circumvent the problem of a missing ground truth.

Here, we generated a benchmark dataset of ~ 12,000 single-cell transcriptomes from eight human cell lines to investigate the performance of scRNA-seq feature selection and clustering approaches. Strikingly, results highlighted a methodology gap for sensitive and specific identification of rare cell types. To fill this gap, we developed a method which we called CellSIUS (Cell Subtype Identification from Upregulated gene Sets). For complex scRNA-seq datasets containing both abundant and rare cell populations, we propose a two-step approach consisting of an initial coarse clustering step followed by CellSIUS. Using synthetic and biological datasets containing rare cell populations, we showed that CellSIUS outperforms existing algorithms in both specificity and selectivity for rare cell type and their transcriptomic signature identification. In addition, and in contrast to existing approaches, CellSIUS simultaneously reveals transcriptomic signatures indicative of rare cell type’s function(s).

To exemplify the use of CellSIUS, we applied the workflow and our two-step clustering approach to complex biological data. We profiled the gene expression of 4857 human pluripotent stem cell (hPSC)-derived cortical neurons generated by a 3D spheroid differentiation protocol. Analysis of this in vitro model of corticogenesis revealed distinct progenitor, neuronal, and glial populations consistent with developing human telencephalon. Trajectory analysis identified a lineage bifurcation point between Cajal-Retzius cells and layer V/VI cortical neurons, which was not clearly demonstrated in other in vitro hPSC models of corticogenesis [23–26]. Importantly, CellSIUS revealed known as well as novel rare cell populations that differ by migratory, metabolic, or cell cycle status. These include a rare choroid plexus (CP) lineage, a population that was either not detected, or detected only partly by existing approaches for rare cell type identification. We experimentally validated the presence of CP neuroepithelia in our 3D cortical spheroid cultures by confocal microscopy and validated the CP-specific signature gene list output from CellSIUS using primary pre-natal human data. For the CP lineage in particular and other identified rare cell populations in general, the signature gene lists output from CellSIUS provide the means to isolate these populations for in vitro propagation and characterization of their role in neurological disorders.

## Results

### Investigation of feature selection and clustering approaches for scRNA-seq data reveals a methodology gap for the detection of rare cell populations

**Fig. 1:**
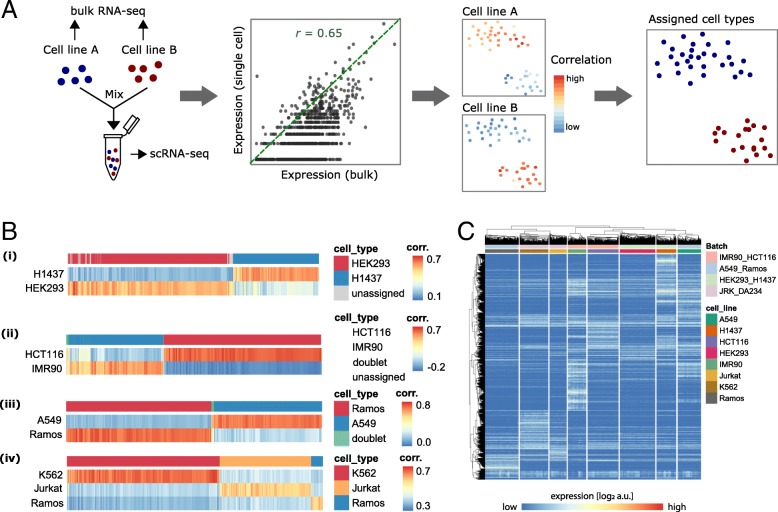
Generation of a scRNA-seq dataset with known cellular composition. **a** Schematic illustration of the experimental setup. Eight human cell lines were individually profiled by bulk RNA-seq and mixed in four batches containing mixtures of two or three cell lines each for scRNA-seq profiling. Correlation of the single-cell to bulk expression profiles was used for cell type assignment as described in the Methods section. **b** Visualization of correlations between single-cell and bulk expression profiles for each batch. The top row represents cell type assignment. Single cells were assigned to the cell type correlating most with their expression profile as described in the Methods section. Cells with *z*-scored correlations below 0.2 were not assigned to any cluster. Cells that correlate strongly with more than one bulk expression profile likely represent doublets and were excluded from future analyses. **c** Heatmap of gene expression values, clustered by their Pearson’s correlation across rows (genes) and columns (cells). The color bars indicate the cell type and the corresponding batch. Only the top 10% genes selected by NBDrop are shown

To assess and compare the performance of some of the most recent and widely used feature selection and clustering methodologies for scRNA-seq data, we generated a scRNA-seq dataset with known cellular composition generated from mixtures of eight human cell lines. To this end, a total of ~ 12,000 cells from eight human cell lines (A549, H1437, HCT116, HEK293, IMR90, Jurkat, K562, and Ramos) were sequenced using the 10X Genomics Chromium platform [3]. Cells were processed in batches containing mixtures of two or three cell lines each. One of the cell lines was present in two separate batches and indicated that technical batch effects were minor as compared to the biological variability (Fig. 1). To infer cell type identity, we profiled each cell line individually using bulk RNA sequencing. Correlation of the single-cell to bulk expression profiles was used for cell type assignment as described in the “Methods” section (Fig. 1a, b). Cells that did not pass quality control (QC) or could not be unambiguously assigned to a cell line (614 cells, ~ 5%) were discarded, leaving 11,678 cells of known cell type (Fig. 1c and Additional file 1: Figure S1, Table S1).

**Fig. 2:**
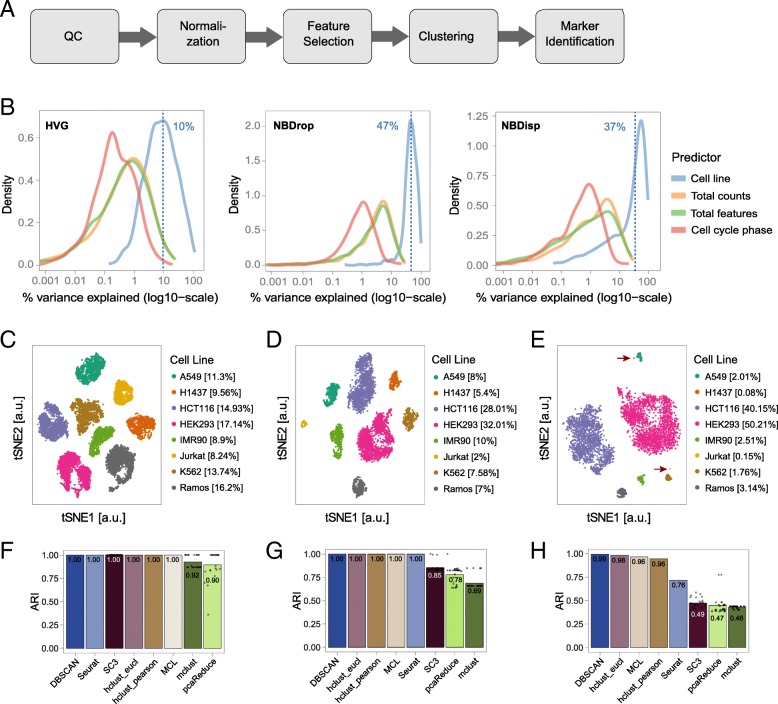
Performance assessment of feature selection and clustering methods. **a** Overview of the computational analysis workflow. **b** Benchmarking of feature selection methods. In each case, the top 10% of features were selected using either a mean-variance trend to find highly variable genes (HVG, left) or a depth-adjusted negative binomial model (DANB) followed by selecting genes with unexpected dropout rates (NBDrop, middle) or dispersions (NBDisp, right). Plots show the percentage of variance explained by each of the four predictors to the total observed variance: cell line, total counts per cell, total detected features per cell, and predicted cell cycle phase. The blue dashed line indicates the average for the predictor cell line. **c**–**e** tSNE projections of the full dataset (**c**) and two sub-sampled datasets with unequal proportions between different cell lines (**d**, **e**). **f**–**h** Comparison of clustering assignments by different methods on the full dataset (**f**), subset 1 (**g**), and subset 2 (**h**). Stochastic methods (SC3, mclust, pcaReduce) were run 25 times. Bars and indicated values represent mean adjusted rand index (ARI), and dots correspond to results from individual runs. All other methods are deterministic and were run only once

We assembled a modular workflow for the analysis of scRNA-seq data (Fig. 2a). The quality control, normalization, and marker gene identification modules were based on recent publications and described in methods. For a data-driven choice of the most appropriate feature selection method upstream of the clustering module, we compared methods using either a mean-variance trend to find highly variable genes (HVG, [27]) or a depth-adjusted negative binomial model (DANB [28]) for selection of genes with unexpected dropout rates (NBDrop) or dispersions (NBDisp). Using linear modeling as implemented in the plotExplanatoryVariables function from scater [29], we quantified the influence of these feature selection methods on the contribution of four predictors to the total observed variance: cell line, total counts per cell, total detected features per cell, and predicted cell cycle phase (Fig. 2b). Results highlighted that (i) for HVG selected genes, cell line accounted for 10% of the total variance only; (ii) for NBDisp and NBDrop selected genes, the percentage of total variance explained by cell line increased to 37% and 47%, respectively, with half of the selected features common to both methods; (iii) genes selected only by NBDisp were generally low expressed (data not shown), highlighting a drawback of variance-based feature selection [28]; and (iv) NBDrop selected features showed an increased contribution of library size (i.e., total detected features and total counts per cell) to the total variance. For our benchmark dataset, the number of total features co-varied with cell type and cell cycle indicating that library size is partially dependent on the cell line (Additional file 1: Figure S1), and thus determined by both technical and biological factors. Therefore, and because in our benchmark dataset, the genes selected by NBDrop explained more cell-line-based variance, we compared some of the most recent or widely used clustering methods after feature selection using NBDrop.

For the clustering module, we investigated seven unsupervised clustering methods for scRNA-seq data (SC3 [15], Seurat [1], pcaReduce, hclust [30], mclust [31], DBSCAN [32], MCL [33, 34], Additional file 1: Table S2) by in silico subsampling of our dataset of known composition in two subsets with different cell type proportions (later referred to as subset 1 and subset 2, Fig. 2c–e, Additional file 1: Table S1). Subset 1 consisted of 4999 cells from eight cell types with abundance varying between 2 and 32%. Subset 2 consisted of 3989 cells with two major cell populations including 90% of all cells of this subset, four medium to low abundant (between 1% and 5%), and two rarer cell types with abundances below 1%, containing 3 (0.08%) and 6 (0.15%) cells, respectively. We applied each clustering method to the complete dataset as well as to both subsets, using principal component analysis (PCA) [35, 36] to project the original expression values to vectors in a lower dimensional space and calculating all distances based on these projections. For all clustering methods, we adjusted parameters such that they resulted in the expected number of 8 clusters. We then assessed the quality of the classification by calculating the adjusted Rand index (ARI) [37] between assignment and true cell line annotation.

On the full dataset, most methods resulted in a perfect assignment (Fig. 2f) with only two of the stochastic methods—pcaReduce and mclust—yielding a lower average ARI of 0.90 and 0.92. In contrast, on subset 1, where cell type proportions were no longer equal, *k*-means-based methods and mclust failed to identify the different cell types correctly and resulted in average ARI of 0.85 (SC3), 0.78 (pcaReduce), and 0.69 (mclust) (Fig. 1g). On subset 2, all methods failed to correctly identify rarer (6 cells, 0.16% of total cells) cell types (Fig. 1h). DBSCAN achieved the highest ARI (0.99) classifying rare cells as outliers (“border points”). All other methods merged rare cells with clusters of abundant cell types resulting in lower ARI of 0.98 (hclust on Euclidean distance), 0.96 (MCL), 0.96 (hclust on correlation distance), and 0.76 (Seurat).

In conclusion, and consistently with a recent review describing the challenges in unsupervised clustering of single-cell RNA-seq data [16], our results showed that most clustering methods performed well in identifying populations defined by more than 2% of total cells. Yet, none of the methods could identify rarer populations, highlighting the need for dedicated tools tailored to detecting rare cell types.

### Development of CellSIUS for rare cell population identification and characterization

**Fig. 3:**
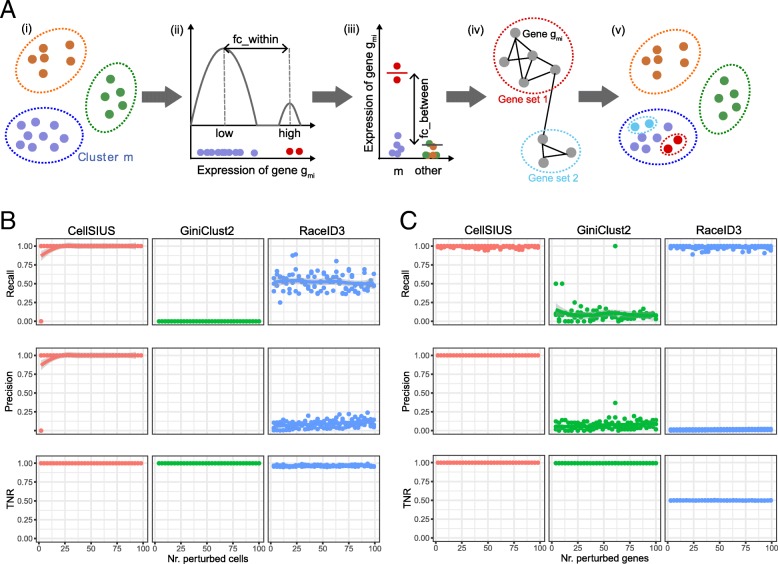
Development and benchmarking of CellSIUS. **a** Schematic overview of CellSIUS. Starting from an initial assignment of N cells in M clusters (i), within each cluster, genes with a bimodal distribution are identified (ii) and only genes with cluster-specific expression are retained (iii). Among the candidate genes, sets with correlated expression patterns are identified by graph-based clustering (iv). Cells are assigned to subgroups based on their average expression of each gene set (v). **b**, **c** Performance comparison of CellSIUS to GiniClust2 and RaceID3 in detecting cells from sub-clusters and their signatures. **b** Recall, precision, and true negative rate (TNR) with regard to the detection of rare cells in synthetic data when varying the number of rare cells from 2 (0.2%) to 100 (10%) **c** Recall, precision, and true negative rate (TNR) with regard to the detection of outlier genes (gene signature) in synthetic data when varying and the number of signature genes from 2 to 100

To overcome the abovementioned limitations, we developed a novel method to identify rare cell populations which we called CellSIUS (Cell Subtype Identification from Upregulated gene Sets). CellSIUS takes as input the expression values of *N* cells grouped into *M* clusters (Fig. 3a). For each cluster *C*_*m*_, candidate marker genes *g*_*m1*_, *g*_*m2*_, …, *g*_*mj*_ that exhibit a bimodal distribution of expression values with a fold change above a certain threshold (fc_within) across all cells within *C*_*m*_ are identified by one-dimensional *k*-means clustering (with *k* = 2). For each candidate gene *g*_*mi*_, the mean expression in the second mode is then compared to this gene’s mean expression level outside *C*_*m*_ (fc_between), considering only cells that have non-zero expression of *g*_*mi*_ to avoid biases arising from stochastic zeroes. Only genes with significantly higher expression within the second mode of *C*_*m*_ (by default, at least a twofold difference in mean expression) are retained. For these remaining cluster-specific candidate marker genes, gene sets with correlated expression patterns are identified using the graph-based clustering algorithm MCL. MCL does not require a pre-specified number of clusters and works on the gene correlation network derived from single-cell RNAseq data and detects communities in this network. These (gene) communities are guaranteed to contain genes that are co-expressed, by design. In contrast, in a *k*-means clustering with a pre-specified *k*, we cannot be sure that all genes within all clusters are co-expressed to the same degree: genes are assigned the closest centroid, but this is only a relative measure. Thus, by using communities of a gene correlation network, with a pre-specified correlation threshold, we can be sure that those communities (if such exist) satisfy the criteria of containing correlated genes. In a last step, cells within each cluster *C*_*m*_ are assigned to subgroups by one-dimensional *k*-means clustering of their average expression of each gene set.

The overall idea behind CellSIUS is similar to RaceID3 [38] and GiniClust2 [19], two recent methods for the identification of rare cell types in scRNA-seq datasets. All of these algorithms combine a global clustering with a second assignment method tailored to the identification of rare cell types. However, in contrast to existing methods, CellSIUS requires candidate marker genes to be cluster specific, and therefore, we hypothesized that our method will be more specific and less sensitive to genes that co-vary with confounders such as the total number of detected genes per cell. To overcome biases associated to the high dropout rates in scRNA-seq, CellSIUS considers only cells that have non-zero expression for the selected marker genes. Finally, in contrast to both RaceID3 and GiniClust2, CellSIUS directly returns a gene signature for each of the new cell subpopulations recovered.

### CellSIUS outperforms existing algorithms in the identification of rare cell populations

We first compared CellSIUS performance to RaceID3 [38] and GiniClust2 [19] using a synthetic dataset. Briefly, we used the expression values of 1000 K562 cells from our dataset to estimate the parameters for the simulation and generated two homogeneous populations of 500 cells (later referred to as clusters 1 and 2). We confirmed the mean-variance and mean-dropout relationships, library sizes, and percentage of zero counts per cells and per gene were similar to the underlying real data (Additional file 1: Figure S2a-f). For this data, both CellSIUS and GiniClust correctly identified the two predefined clusters whereas RaceID3 detected a large number of false positives (Additional file 1: Figure S2 g).

We then assessed each algorithm’s ability to detect an increasingly rare cell type by adding between 2 and 100 (0.2–10% of the cluster size) cells of a third type to the two homogenous populations described above. This new synthetic cell type was generated by increasing the log2 expression values of 20 randomly selected genes by an average of 2.5.

We compared (i) recall as the fraction of rare cells correctly assigned to new clusters, i.e., the number of correctly identified rare cells divided by the total number of rare cells; (ii) precision as the fraction of true rare cells among all cells not assigned to the two main clusters; and (iii) true negative rate (TNR) as the fraction of abundant cells that were correctly assigned to the two main clusters. To enable a more direct comparison between the methods, benchmarking analyses were carried out with a predefined initial clustering for all approaches. CellSIUS had a recall of 1 for rare cell populations consisting of more than 2 cells. In contrast GiniClust2 did not identify any rare cell populations and RaceID3 recalled only ~ 50% of true positives (Fig. 3b, top panel). Additionally, CellSIUS exhibited a TNR of 1.0 and thus a precision of 1.0 (except in the one case where no true positives were recovered). While GiniClust2’s TNR was also 1.0, the precision could not be defined due to the lack of identification of true and false positives. RaceID3 had a low TNR (mean = 0.95, sd = 0.01), resulting in low precision (mean = 0.1, sd = 0.1) (Fig. 3b, middle and bottom panel). We then repeated this comparison for the identification of signature genes. To this end, we generated a second set of populations. Briefly, the number of rare cells was fixed at 20 (~ 2% of total cells), and we increased the log2 expression values of between 2 and 100 genes by 2.5 on average. We compared (i) recall, (ii) precision, and (iii) TNR as above but with respect to genes. In comparison to CellSIUS, GiniClust2 showed a poor performance (Fig. 3c, top panel), consistent with failing to detect rare cell population. In contrast, RaceID3 performed slightly better than CellSIUS in terms of recall, however, with a precision cost. Whereas both precision and TNR were 1.0 for CellSIUS, RaceID3 had a low TNR (0.5) and consequently a low precision (mean = 0.012, sd = 0.007) (Fig. 3c, top and bottom panels).

To systematically investigate the stability of CellSIUS’ output to parameter changes, we repeated the above-described analysis when varying fc_within, fc_between and corr_cutoff (Additional file 1: Figure S3; Methods). Results that highlighted the stability of both sensitivity and specificity are across a wide range of parameters.

In summary, using synthetic data, we showed an increased sensitivity and specificity of our algorithm for rare cell type identification and outlier gene identification compared to GiniClust2 and RaceID3 (Fig. 3b, c) and demonstrated robustness to parameter choices (Additional file 1: Figure S3).

**Fig. 4:**
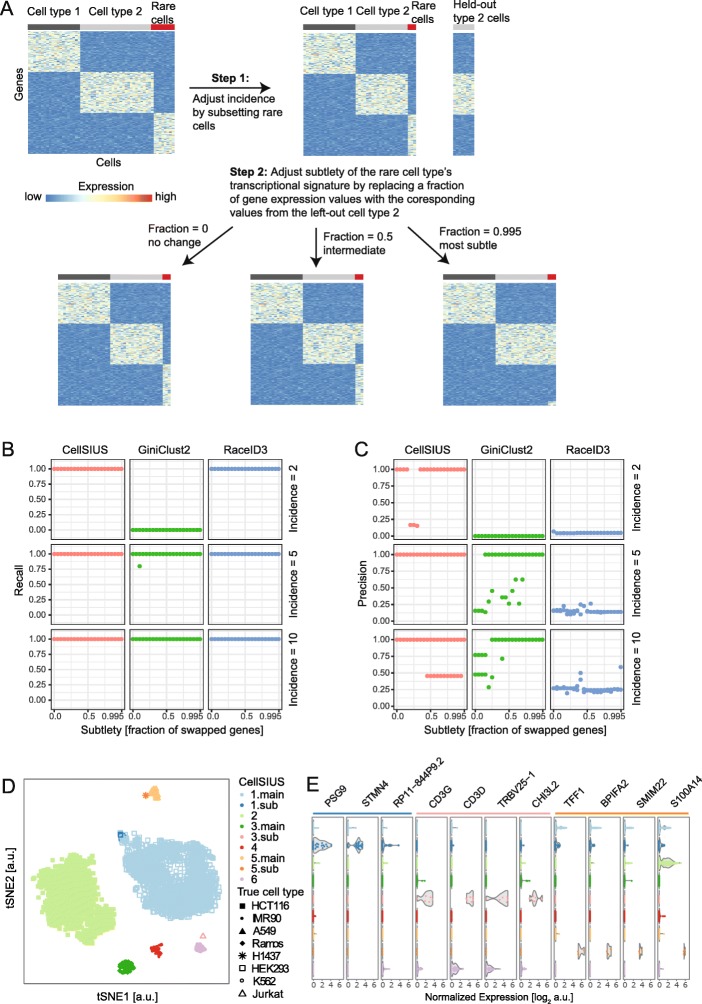
CellSIUS benchmarking on cell line data. **a** Schematic overview of dataset perturbations. Starting from a dataset containing three cell types (abundant cell type 1, abundant cell type 2, and rare cell type), we first generated a defined number of rare cells by subsampling. In addition, we partitioned the type 2 cells in two, leaving out 25 cells from the dataset for later use. Next, we adjusted the subtlety of the transcriptional difference between the rare cells and their closest neighbor (cell type 2) by swapping a fraction of gene expression values in the type 2 cells with the corresponding value in the left-out rare cells. We then pre-defined an initial cluster assignment as cluster 1 = type 1, cluster 2 = the union of type 2 and rare cells and assessed whether different algorithms for detecting rare cell types are able to correctly classify the rare cells as such. **b**, **c** Comparison of CellSIUS to GiniClust2 and RaceID3 for varying incidence of the rare cell type and varying subtlety of the transcriptional signature here, we used 100 HEK293 cells as type 1, 100 Ramos cells as type 2, and up to 10 Jurkat cells as the rare cell type and we swapped between 0 and 99.5% of gene expression values. For each algorithm, we assessed the recall (**b**), i.e., the fraction of correctly identified rare cells, and precision (**c**), i.e., the probability that a cell which is classified as rare is actually a rare cell. **d** tSNE projection of subset 2 of the cell line dataset, colored by CellSIUS assignment. Cluster numbers correspond to the main clusters identified by MCL, clusters labeled x.sub indicate the CellSIUS subgroups. Symbols correspond to the cell line annotation. **e** Violin plot showing the main markers identified by CellSIUS, grouped by cluster

We next benchmarked CellSIUS’ specificity and selectivity using our dataset of known cell composition, randomly subsampling 100 HEK293 cells and 125 Ramos cells, and including 2, 5, or 10 Jurkat cells. Only cells assigned to be in cell cycle phase G1 were considered to ensure within-cluster homogeneity. To simulate varying degrees of transcriptional difference between the rare cell type (Jurkat) and its closest more abundant cell type (Ramos), we adapted an approach recently presented by Crow et al. [39] (Fig. 4a). Briefly, from the initial dataset, 25 Ramos cells were held out. Subsequently, an increasing fraction of gene expression values in the Jurkat cells were replaced by the respective values in the held out Ramos cells, thus diluting the Jurkat-specific gene expression profile and making the Jurkat cells more and more similar to Ramos. Using this approach, we generated datasets with two equally sized abundant populations (HEK293 and Ramos, 100 cells each) and one rare population (Jurkat, varying between 2, 5, and 10 cells). We predefined two initial clusters: cluster 1 contained all HEK293 cells and cluster 2 combined the two lymphomas (Ramos and Jurkat).

We then tested the ability of CellSIUS, RaceID3, and GiniClust2 to identify rare cell types for varying incidence (i.e., total number of rare cells) and subtlety (i.e., fraction of Jurkat genes replaced by Ramos genes). We assessed the recall (Fig. 4b) and precision (Fig. 4c) as above. Results showed a high sensitivity of all three methods for very subtle transcriptional signatures (99.5% of genes replaced, corresponding to 230 unperturbed genes) and low incidence (down to two cells except for GiniClust2). However, CellSIUS exhibited high precision (88.4% on average), in comparison to GiniClust2 (51.6% on average) and RaceID3 (15.6% on average).

Having shown that CellSIUS is more sensitive and specific for the identification of rare cell types and outlier genes using synthetic and simulated biological data, we tested its ability to reveal transcriptomic signatures indicative of rare cell type’s function(s). We applied CellSIUS to subset 2 of our dataset of known composition (Additional file 1: Table S1) with 6 clusters predefined using MCL (Fig. 4d). CellSIUS identified three subgroups (Jurkat, H1437, and a small subgroup of IMR90 cells) within the 6 initial clusters characterized by upregulation of three or more genes (Fig. 4e). Notably, the two strongest signatures were obtained for the two subgroups corresponding to Jurkat and H1437 cells with top marker genes consistent with previous knowledge: *CD3G* and *CD3D*, both of which are known T cell markers [40] being the top markers for Jurkat (T cell lymphoma), and *TFF1* and *BPIFA2,* both shown to function in the respiratory tract [41, 42] being the top markers for H1437 (lung adenocarcinoma, epithelial/glandular cell type).

Taken together, these results show that CellSIUS outperforms existing methods in identifying rare cell populations and outlier genes from both synthetic and biological data. In addition, CellSIUS simultaneously reveals transcriptomic signatures indicative of rare cell type’s function.

### Application to hPSC-derived cortical neurons generated by 3D spheroid directed-differentiation approach

As a proof of concept, we applied our two-step approach consisting of an initial coarse clustering step followed by CellSIUS to a high-quality scRNA-seq dataset of 4857 hPSC-derived cortical neurons generated by a 3D cortical spheroid differentiation protocol generated using the 10X Genomics Chromium platform [3] (Additional file 1: Figure S4a and Table S3; see the “Methods” section). During this in vitro differentiation process, hPSCs are expected to commit to definitive neuroepithelia, restrict to dorsal telencephalic identity, and generate neocortical progenitors (NP), Cajal-Retzius (CR) cells, EOMES^+^ intermediate progenitors (IP), layer V/VI cortical excitatory neurons (N), and outer radial-glia (oRG) (Additional file 1: Figure S4b). We confirmed that our 3D spheroid protocol generates cortical neurons with expected transcriptional identity that continue to mature upon platedown with expression of synaptic markers and features of neuronal connectivity at network level [43] (Additional file 1: Figure S4c, d, e, and see the “Methods” section).

**Fig. 5:**
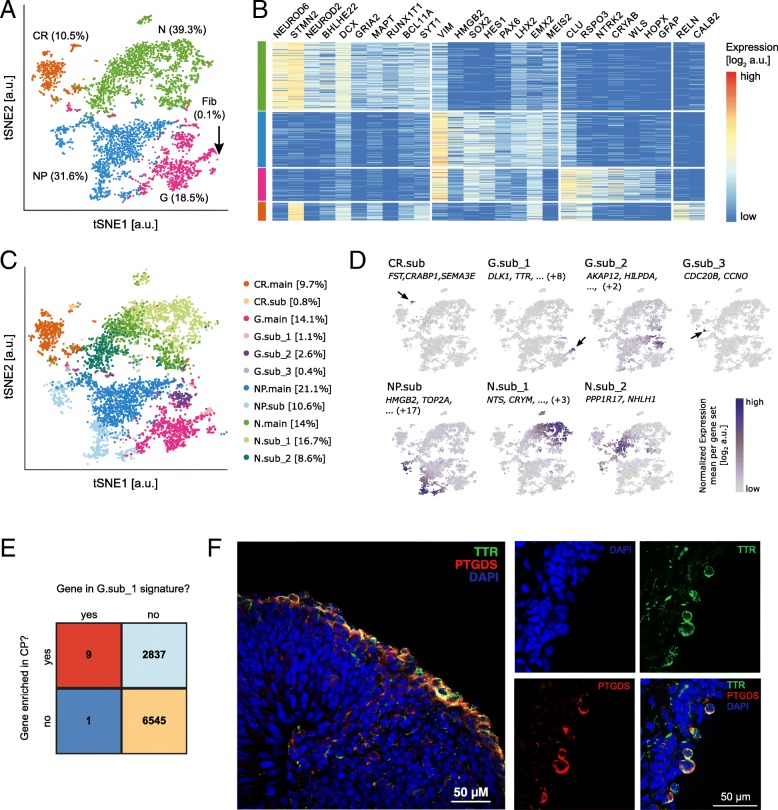
Characterization of hPSC-derived cortical excitatory neurons by scRNA-seq. **a** tSNE projection of 4857 single-cell transcriptomes of hPSC-derived neuronal cell types after 86 days of differentiation. Unsupervised clustering using MCL groups cells into four major classes: Neurons (N), neuroepithelial progenitors (NP), mixed glial cells (G), and Cajal-Retzius cells (CR). In addition, a small population of fibroblasts (Fib) is identified. **b** The identified cell populations are characterized by expression of known markers for the expected cell types. Expression values are shown as log2 (normalized UMI counts + 1). **c** tSNE projection, colored by CellSIUS assignment. Main clusters are denoted .main, subclusters .sub. **d** Mean expression of each marker gene set identified by CellSIUS, projected onto the same tSNE map as shown in **a**. The top markers are indicated for each gene sets; numbers in brackets refer to how many additional genes are part of the marker gene set. **e** Comparison of the gene signature uncovered by CellSIUS to genes found to be enriched (*p* < 0.05) in choroid plexus of the fourth ventricle according to harmonizome [47, 48]. **f** Single optical sections of neurosphere cryosections acquired by confocal microscopy showing co-localization of TTR and PTGDS in cells predominantly on the periphery of neurospheres (panel left—composite image of a neurosphere; panels right—split images from a different neurosphere)

Initial coarse-grained clustering using MCL identified four major groups of cells that specifically express known markers for NPs [44], mixed glial cells (G), CR cells [45], and neurons (N) [46] (Fig. 5a, b). A small population of contaminating fibroblasts (0.1% of total cells) was removed from the dataset for downstream analyses. CR cells expressed *DCX*, *CALB2*, *STMN2*, and *MAPT* consistently with developing mouse and human cortex (Fig. 5b) [49–51]. The robust expression of *FOXG1* in the general population (Additional file 1: Figure S5a) and the expression of *PAX6*, *EMX2*, and *LHX2* in NPs (Fig. 5b) indicated our differentiation protocol mainly generates cells with dorsal telencephalic identity [52].

Applying CellSIUS to this data identified 7 subpopulations (Fig. 5c, d). Notably, within the mixed glial cells (G), CellSIUS identified a rare subgroup (1.1% of total population, G.sub_1) characterized by a signature of 10 genes. Nine of those *((TRPM3, PTGDS, TTR, CXCL14, HTR2C, WIF1, IGFBP7, MT1E, DLK1*) are known to be enriched in primary pre-natal human choroid plexus (CP) (Fig. 5e) compared to the other tissues from the developing human cortex (harmonizome database [47, 48] using a cutoff of 1.3 for the standardized value, corresponding to a Benjamini-Hochberg-corrected *p* adjusted < 0.05). This G.sub_1 population is therefore consistent with the formation of CP, a secretory neuroepithelial tissue that produces cerebrospinal fluid (CSF) and that has multiple origins along the rostro-caudal axis of the developing nervous system including the dorsal telencephalic midline [53]. We further validated the presence of CP neuroepithelia in our 3D human cortical cultures by confocal microscopy analysis. Using neurosphere cryosections, we demonstrated co-localization of canonical CP marker transthyretin (TTR) with prostaglandin D2 synthase (PTGDS), another CP enriched protein described in primary mouse and human tissue, in a limited number of cells located almost exclusively on the periphery of neurospheres (Fig. 5f). Collectively, these results suggest that the 3D spheroid human cortical differentiation protocol described here can generate developmentally relevant cell types and that CellSIUS can identify rare cell populations within the heterogeneity and complexity of stem cell-based models.

CellSIUS identified a second subgroup in the mixed glial cells (G) characterized by high expression levels of glycolytic enzymes (G.sub_2, 2.6%) (Fig. 5c, d and Additional file 1: Figure S6a). Analysis between G.sub_2 and the rest of the G cells revealed upregulation of *HOPX, PTPRZ1, CLU, BCAN, ID4,* and *TTYH1* in the main group, a transcriptional signature consistent with developing human outer radial glia (oRG) [54]*,* (Additional file 1: Figure S6a Additional file 2: Table S4). oRG cells also upregulated mitochondrial genes (Additional file 2: Table S4) that are crucial for oxidative phosphorylation, highlighting the metabolic difference between these two groups. We hypothesize the G.sub_2 subgroup to be a progenitor population that is located closer to the hypoxic interior of neurospheres, a common feature of the 3D spheroid differentiation protocols.

In addition, CellSIUS identified a subgroup of NP cells (NP.sub, 10.6%) defined by upregulation of cell-cycle-related genes such as *HMGB2, TOP2A*, and *MKI67* (Fig. 5c, d, Additional file 1: Figure S6a) as well as a subgroup of CR cells (CR.sub, 0.8%) characterized by *SEMA3E*, *BTG1*, and *PCDH11X* (Fig. 5b and Additional file 1: Figure S6A) which may represent CR cells at a different stage of migration [55–57].

Finally, CellSIUS revealed a split in the neuronal population (N), identifying 2 groups, N.sub_2 (8.6%) and N.sub_1 (16.7%) (Fig. 5c, d, Additional file 1: Figure S6a)*.* In addition to *NHLH1* and *PPP1R17* known to be enriched in immature neurons [54], N.sub_2 expressed *EOMES* (Additional file 1: Figure S5b), a well-characterized marker of cortical intermediate progenitors [46, 54] that give rise to TBR1^+^ cortical neurons (Additional file 1: Figure S5c) and is likely a mixed population of intermediate progenitors and immature neurons. In contrast, markers identified by CellSIUS for the N.sub_1 neuronal population were unexpected. Although co-expression of *FEZF2*, *CRYM*, *PCDH17,* and *RUNX1T1* in this cortical neuronal population is consistent with recent scRNA-seq data from the developing human cortex (Additional file 1: Figure S7b, EN-V1–1: Early-born deep-layer/sub-plate excitatory neurons, EN-PFC1: Early-born deep-layer/sub-plate excitatory neurons prefrontal cortex), robust *NTS* expression in developing cortical neurons has not been reported so far to the best of our knowledge. The expression of *FEZF2* (Additional file 1: Figure S5d) in this culture which is consistent with the general dorsal telencephalic identity of these cells and co-expression of *FEZF2* and *BCL11B* (CTIP2) in this particular post-mitotic neuronal sub-population (Additional file 1: Figure S5d-e) could suggest patterning towards cortico-spinal motor neurons (CSMNs). However, the presence of *NTS,* which encodes a 13-amino acid neuropeptide called neurotensin highly expressed in the hypothalamus and amygdala, is not in line with the overall transcriptional identity as discussed above. Analysis of a recently published scRNA-seq dataset from different regions and developmental stages of the human cortex [46] revealed that only a few cells derived from the fetal primary visual cortex (age 13 pcw) express *NTS* (Additional file 1: Figure S7). The limited number of cells in our dataset limits any firm conclusions.

**Fig. 6:**
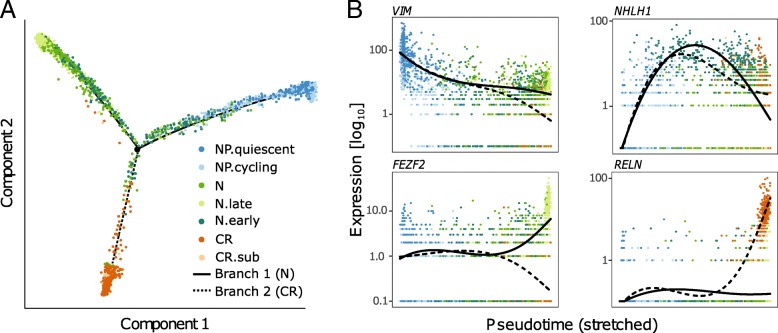
Monocle analysis of the NP, N, and CR cluster. **a** Consistent with the subgroup assignment by CellSIUS, monocle orders cells on a trajectory from NP via immature neurons (N_early) to either mature N or CR cells. **b** Gene expression along pseudotime. Shown is a marker for NPs (VIM), immature neurons (NHLH1), N.sub_2 (FEZF2), and CR cells (RELN)

To further characterize the transition from progenitors to the two different neuronal cell types (CR cells and all N populations), we applied Monocle for trajectory analysis to a subset of the cells corresponding to these three identities. This analysis revealed a tree with two branches (Fig. 6a). As expected, cells progress from the tree root which is composed of progenitors via the NHLH1^high^/PPP1R17^high^ population towards either N (branch 1) or CR cells (branch 2). Along the trajectory, the NP marker *VIM* decreases gradually whereas *NHLH1* increases up to the branch point, then decreases again (Fig. 6b). The CR branch ends with cells expressing high levels of *RELN*, and the N branch is characterized by gradual increase of *FEZF2* expression and ending in the N.sub_1 population (Fig. 6b). Notably, at the very tip of this branch, we also find a very small number of cells expressing *LDB2* and *DIAPH3* which are markers of CSMNs in the mouse [58]. It is plausible that, given more time, this population may eventually give rise to CSMNs with a more defined transcriptional signature.

### Comparison of CellSIUS, RaceID3, and Giniclust2 performance for rare cell type identification in hPSC-derived cortical neurons

To get an understanding of how CellSIUS, GiniClust2, and RaceID3 differ in the identification of rare cell types from a complex dataset, we compared their output when run on the cortical neuron datasets. Because a classic benchmarking is not possible here due to the lack of a ground truth, we instead focus on comparing the ability of each algorithm to reveal experimentally validated signatures or cell types known from literature. As before, we used the same initial of 4 main clusters identified by MCL (Fig. 5a) for all algorithms. GiniClust2 resulted in a total of 20 clusters. The main differences between GiniClust2 and CellSIUS (Additional file 1: Figure S6b) results can be summarized as follows: (i) GiniClust2 generated clusters that merge major known cell types (for example cluster 14 merges G, G.sub_1 (=CP), G.sub_2, N, N.sub_1 (late neurons) and N.sub_2 (early neurons)), and (ii) GiniClust2 did not detect CP (G.Sub_1), cycling NPs (NP.sub) nor the well-described immature neurons (N.sub_2).

RaceID3 with default settings resulted in a total of > 50 clusters, consistent with the high false-positive rate observed with synthetic and cell line data. With a more stringent outlier probability cutoff (10^−20^), RaceID3 identified 10 clusters with a similar overall assignment to CellSIUS (Additional file 1: Figure S6c). However, if RaceID3 did partly detect CP (G.Sub_1), it also split the CP cluster identified by CellSIUS across several other clusters with the majority of cells assigned to either cluster 3 (19 CP together with 4 other cells) or cluster 5 (mixed with a large number of G, N, and NP cells). The CP markers *PTGDS* and *TTR* are co-expressed in 49/53 CP cells identified by CellSIUS but only in 19/54 CP cells identified by RaceID3 suggesting that RaceID3 incorrectly assigned most of the CP cells to a merged G/NP/N cluster. In addition, and similarly to GiniClust2, RaceID3 did identify neither cycling NPs (NP.sub) nor the above-described progenitors and immature neurons population (N.sub_2).

In summary, these results indicate superior performance with regard to specificity and sensitivity of CellSIUS compared to other approaches when applied to the complex and heterogeneous data generated here and demonstrate the algorithm’s ability to identify rare populations within major cell types that differ by their metabolic state, cell cycle phase, or migratory state.

## Discussion

We generated a benchmark dataset of ~ 12,000 single-cell transcriptomes from 8 cell lines to compare the performance of some of the most recent and widely used scRNA-seq feature selection and clustering approaches. Our findings suggest that in our dataset, for unsupervised feature selection, the DANB methods implemented in the M3Drop package outperformed HVG. While all clustering methods tested performed equally well on data with balanced and abundant cell populations, *k*-means and model-based methods performed poorly on subsampled datasets with unequal cell type proportions, typically splitting clusters containing many cells while merging those containing few cells. This is likely a consequence of feature selection and PCA-based dimensionality reduction prior to clustering where these methods select or assign weights to genes based on mean expression and variance across the whole cell population, which are both low if a gene is specifically expressed in a small subset of cells only.

In contrast, hclust in combination with dynamicTreeCut, MCL, and DBSCAN resulted in accurate cluster assignments across all subsampled datasets. Strikingly, none of the methods we tested was able to identify rare cell types (< 1% in this dataset). It is worth noting that although DBSCAN does classify rare cell types as border points, it did however not reliably identify these populations for two reasons: (i) additional cells that did not belong to the rare populations are also classified as border points; (ii) DBSCAN does not perform well if there are points connecting clusters, which is often the case in scRNA-seq datasets. In summary, our comparison of clustering methods is consistent with a recent review describing the challenges in unsupervised clustering of single-cell RNA-seq data [16], highlighting the methodology gap for detecting rare cell types.

To overcome these limitations, we developed CellSIUS, a novel algorithm that takes initial coarse clusters as input and identifies rare cell subtypes based on correlated gene sets specific to subpopulations. Based on our comparison of clustering methods above, we used MCL as our default clustering method: MCL showed a high accuracy in the comparison to other methods, requires fewer parameter choices than hclust for defining the number of clusters, and, unlike DBSCAN, assigns all points to clusters.

The overall idea behind CellSIUS is similar to RaceID3 [38] and GiniClust2 [19], two recent methods for the identification of rare cell types in scRNA-seq datasets. All of these algorithms combine a global clustering with a second assignment method which is tailored to finding rare cell types. There are however important differences between the approaches which are at the basis of CellSIUS’ superior performance for both rare cell type as well as outlier genes’ identification in terms of specificity and selectivity.

RaceID3’s initial step is a *k*-medoids clustering, followed by outlier cell identification in each cluster in four steps: (i) calibration of a background model of gene expression by fitting a negative binomial distribution to the mean and variance of each gene in each cluster; (ii) identification of outlier cells by calculating for each gene and each cell the probability of observing this expression value under the assumption of the background model; (iii) merging of potential outlier cells into new clusters based on the similarity of their gene expression; and (iv) definition of new cluster centers for both the original and the outlier clusters. In a final step, cells are assigned to the cluster they are closest to. In contrast to CellSIUS, RaceID3 does not require the outlier genes to be cluster specific; consequently, it may select genes that co-vary with technical confounders such as the total number of detected genes per cell. In addition, whereas CellSIUS only considers subcluster-specific genes to assign cells to final clusters, the final cluster assignment in RaceID3 is done based on the similarity of each cell’s whole transcriptomic signature to each cluster center. In cases where the distance between the outlier cluster and neighboring clusters is small, this leads to a high number of false positives, with many cells initially not identified as outliers being merged into the nearest outlier cluster.

GiniClust2 runs two independent clustering steps on the same data. The first clustering aims at capturing global structure of the data by running a *k*-means clustering on the expression of genes with a high Fano factor. This is motivated by the fact that a high Fano factor is associated with genes that are differentially expressed between abundant cell types. The second clustering is performed by running a density-based clustering on genes with a high Gini index which is typically associated with genes being differentially expressed between rare and abundant cells. In a final step, the results of both clustering are merged based on a weighted consensus association. The main differences to CellSIUS are as follows: (i) the selection of the genes for the rare cell type assignment is performed using a global metric (i.e., the Gini coefficient across the whole dataset), whereas CellSIUS takes into account the information on the global clustering (e.g., considers only cluster-specific genes), and (ii) the final assignment is a weighted average of the results from both clustering steps, whereas we use a two-step approach consisting of an initial coarse clustering step followed by CellSIUS for the identification of rare cell types and outlier genes.

Enforcing gene signatures to be cluster-specific comes with the promise to overcome some technical biases, e.g., different number of detected genes between cells, differences in the total number of counts per cell or normalization artifacts. For example, normalization may lead to artificially high counts for abundant transcripts in cells that have overall few detected genes. These genes are, however, present across different clusters and would therefore not be considered a valid signature. While restricting to cluster-specific signatures has the potential to help disentangle technical and biological variability and increase the precision of rare cell type identification, it comes with the limitation of potentially missing rare cell types spread over multiple clusters. This issue could be addressed by iteratively merging the most similar clusters and re-running CellSIUS for each initial cluster definition. A further consideration is CellSIUS’ output sensitivity to initial cluster assignments. In practice, this should only be an issue if there is no clear global structure in the data and cluster assignments are not consistent between different clustering methods and/or parameter settings. In such cases, one could use a consensus assignment from a combination of different clustering assignments.

To exemplify the added value of CellSIUS over existing approaches in a real-world setting, we applied the workflow and our two-step clustering approach to a complex biological dataset consisting of hPSC-derived neurons. We identified major neural cell types of early human corticogenesis such as cycling and quiescent NPs, *EOMES*^+^ IPs, CR cells, immature and mature neurons with a transcriptional identity indicative of layer V/VI neurons, and oRG. Overall, the transcriptional fingerprint of each major group was in line with a recent scRNA-seq data set from the developing human cortex. CellSIUS analysis also revealed a transcriptional signature in the mature neuronal population that deviates from the expected cortical trajectory, typified by the high expression levels of *NTS* detected in N.sub_1, highlighting the importance of unbiased characterization of hPSC differentiation platforms at single-cell level. Single-cell trajectory analysis of NP, CR, and N cells using Monocle revealed a pseudo-temporal order of progenitors gradually differentiating into neurons, with a lineage split between Cajal-Retzius cells and *FEZF2*^+^ neurons.

Importantly, CellSIUS identified known as well as novel rare cell types within the major groups, such as putative CP (G.sub_1), a population that was either not detected, or detected only partly by existing approaches for rare cell type identification. Single-cell RNA-seq data usually contains a small fraction of doublets, i.e., transcriptomes derived from two or more cells, which could form artifactual clusters. Our results do not indicate the presence of doublet-driven clusters—each subcluster has its own unique markers. In addition, most of the subpopulation signatures represent biological function that is supported by the literature. Finally, we experimentally validated the presence of CP neuroepithelia in our 3D cortical spheroid cultures by confocal microscopy and validated the CP-specific signature gene list identified by CellSIUS using primary pre-natal human data. For the CP lineage in particular and other identified rare cell populations in general, the signature gene lists output from CellSIUS provide the means to isolate these populations for in vitro propagation and characterization of their role in neurological disorders.

## Conclusions

In this study, we present CellSIUS, a novel method to identify and characterize rare cell types from complex scRNA-seq datasets. Benchmarking of CellSIUS on synthetic data and a large dataset with known cell composition generated from 8 human cell lines demonstrated the high sensitivity and specificity of CellSIUS over existing approaches. Characterization of a novel human pluripotent cell differentiation protocol recapitulating deep-layer corticogenesis in vitro using scRNA-seq and CellSIUS revealed previously unrecognized complexities in human stem cell-derived cellular populations. Importantly, CellSIUS enabled identification of known and novel rare cell populations and their signature gene list providing the means to study those populations in vitro in light of their role in health and disease.

## Methods

### Human cell lines

For the benchmarking dataset, 8 different human cell lines from the ATCC biorepository have been used (Table 1). Cell lines were shown to be mycoplasma free using the Mycoalert kit from Lonza.

**Table 1:** Cell lines and culture conditions used in this study

Cell line	Gender	Cell type	Tissue of origin	Obtained from	Culture conditions
A549	M	Alveolar basal epithelial (adherent)	Lung adenocarcinoma	ATCC CCL-185	ATCC F12K (ATCC, P/N 30-2004) +10% FCS (AMIMED, P/N 2-01F36-I).
H1437	M	Epithelial/glandular (adherent)	Lung adenocarcinoma, derived from metastatic site: pleural effusion	ATCC CRL-5872	RPMI (Invitrogen, P/N A1049101) +10% FBS (ATCC, P/N SCRR-30-2020)
HCT116	M	Epithelium-like (adherent)	Colon carcinoma	ATCC CCL-247	ATCC McCoy's 5A (ATCC, P/N 30-2007) + 10% FCS (AMIMED, P/N 2-01F36-I)
HEK293	F	Epithelial (adherent)	Transformed cell line, derived from embryonic kidney	ATCC, P/N CRL-1573	ATCC EMEM (ATCC, P/N 30-2003) +10% FCS (AMIMED, P/N 2-01F36-I)
IMR90	F	Fibroblast (adherent)	Fetal lung	ATCC CRL-186	ATCC EMEM (ATCC, P/N 30-2003) 10% FCS (AMIMED, P/N 2-01F36-I)
Jurkat	M	T cell (suspension)	Childhood T acute lymphoblastic leukemia	ATCC, P/N TIB-152	RPMI (Invitrogen, P/N 61870-044) + 10% FCS (AMIMED, P/N 2-01F36-I)
K562	F	Undifferentiated, lymphoblast with granulocyte/erythrocyte/monocyte characteristics (suspension)	Chronic myelogenous leukemia, BCR-ABL1 positive	ATCC, P/N CRL-1573	RPMI (Invitrogen, P/N 61870-044) + 10% FCS (AMIMED, P/N 2-01F36-I).
Ramos	M	B cell (suspension)	Burkitt’s lymphoma	ATCC, P/N CRL-1596	Batch 3: RPMI (Invitrogen, P/N A1049101) +10% FBS (ATCC, P/N SCRR-30-2020) Batch 4: RPMI (Invitrogen, P/N 61870-044) + 10% FCS (AMIMED, P/N 2-01F36-I)

### Single-cell RNA-sequencing of cell lines

Cellular suspensions were loaded on a 10x Genomics Chromium Single Cell instrument to generate GEMs. Single-cell RNA-seq libraries were prepared using GemCode Single Cell 3′ Gel Bead and Library Kit according to CG00052_SingleCell3’ReagentKitv2UserGuide_RevB. GEM-RT was performed in a Bio-Rad PTC-200 Thermal Cycler with semi-skirted 96-well plate (Eppendorf, P/N 0030 128.605): 53 °C for 45 min and 85 °C for 5 min, held at 4 °C. After RT, GEMs were broken and the single strand cDNA was cleaned up with DynaBeads® MyOne™ Silane Beads (Life Technologies P/N, 37002D). cDNA was amplified using a Bio-Rad PTC-200 Thermal cycler with 0.2-ml 8-strip non-Flex PCR tubes, with flat Caps (STARLAB, P/N I1402–3700): 98 °C for 3 min; cycled 12x: 98 °C for 15 s, 67 °C for 20 s, and 72 °C for 1 min; 72 °C for 1 min; and held at 4 °C. Amplified cDNA product was cleaned up with the SPRIselect Reagent Kit (0.6X SPRI). Indexed sequencing libraries were constructed using the reagents in the Chromium Single Cell 3′ library kit V2 (10x Genomics P/N-120237), following these steps: (1) fragmentation, end-repair and A-tailing; (2) post fragmentation, end-repair, and A-tailing double sided size selection with SPRIselect Reagent Kit (0.6X SPRI and 0.8X SPRI); (3) adaptor ligation; (4) post-ligation cleanups with SPRIselect (0.8X SPRI); (5) sample index PCR using the Chromium Multiplex kit (10x Genomics P/N-120262); (6) post sample index double sided size selection—with SPRIselect Reagent Kit (0.6X SPRI and 0.8X SPRI). The barcode sequencing libraries were quantified using a Qubit 2.0 with a Qubit™ dsDNA HS Assay Kit (Invitrogen P/N Q32854), and the quality of the libraries was performed on a 2100 Bioanalyzer from Agilent using an Agilent High Sensitivity DNA kit (Agilent P/N 5067–4626). Sequencing libraries were loaded at 10 pM on an Illumina HiSeq2500 with 2 × 50 paired-end kits using the following read length: 26 cycles Read1, 8 cycles i7 Index, and 98 cycles Read2. The CellRanger suite (2.0.2) was used to generate the aggregated gene expression matrix from the BCL files generated by the sequencer based on the hg38 Cell Ranger human genome annotation files.

### Bulk RNA-sequencing of cell lines

For each individual cell line, RNA was isolated from 5 × 10^5^ cells using the RNeasy Micro kit (Qiagen, Cat# 74104). The amount of RNA was quantified with the Agilent RNA 6000 Nano Kit (Agilent Technologies, Cat# 5067–1511). RNA sequencing libraries were prepared using the Illumina TruSeq RNA Sample Prep kit v2 and sequenced using the Illumina HiSeq2500 platform. Samples were sequenced to a length of 2 × 76 base-pairs. Read pairs were mapped to the *Homo sapiens* genome (GRCh38) and the human gene transcripts from Ensembl version 87 [59] by using an in-house gene quantification pipeline [60]. Genome and transcript alignments were used to calculate gene counts based on Ensembl gene IDs.

### Differentiation of cortical excitatory neurons from human pluripotent stem cells in suspension

H9-hESCs (WA09) were obtained from WiCell and maintained in TeSR-E8 medium (Stemcell Tech., 05990) on tissue-culture plates coated with vitronectin (Gibco, A14700). hESCs were passaged using ReLeSR (Stemcell Tech., 05873) to dissociate into cell clumps and were replated in E8 plus thiazovivin (Selleckchem, S1459) at 0.2 μM. H9-hESC line was free of mycoplasma and was tested using the Mycoalert detection kit (Lonza).

hESCs were changed to mTesR1 (Stemcell Tech., 85,850) media when they were 70–80% confluent and maintained in mTesR1 for a minimum of 2 days before confluent monolayer of hESCs were neurally converted by changing the media to phase I (Additional file 1**:** Table S5). Seven days post induction, cells were dissociated to single-cell suspension with Accutase (Gibco A1110501), seeded at 1.5E6 cells/mL in spinner flasks with phase II media **(**Additional file 1**:** Table S5) supplemented with 2 μM Thiazovivin and 10 ng/mL FGF2 (Peprotech, 100-18B) (final) and incubated at 37 °C on a micro-stir plate at 40 rpm for 4 days. Media was then changed to phase III (Additional file 1**:** Table S5), and neurospheres were further cultured for 17 days at 60 rpm, changing media 50% twice a week. On day 28, media were changed to phase IV **(**Additional file 1: Table S5) and cultures were maintained 21 more days with 50% media change twice a week. From day 49 onwards, cultures were switched to Ph IV media for maintenance. Neurospheres were dissociated with Papain kit (Worthington) at day 86 for single-cell RNAseq or neuronal platedowns on laminin (Sigma, L2020), fibronectin (Corning, 354,008), and Matrigel (Corning, 354,230) coated plates.

### Characterization of cortical neurons generated by the 3D spheroid protocol

Generation of layer V/VI neuronal populations was confirmed by immuno-fluorescence analysis of D86 cultures upon dissociation and plating, showing robust expression of deep-layer cortical neuronal markers TBR1 and CTIP2 (Additional file 1: Figure S4c). Cortical neurons generated by the 3D spheroid protocol co-cultured with rat glia for 4 weeks were positive for pre- and post-synaptic markers Synaptophysin I and PSD-95 (Additional file 1: Figure S4d). Calcium imaging by FDSS 7000EX platform demonstrated spontaneous intracellular calcium oscillations, indicating that spontaneous firing was synchronized between the majority of the cortical neurons in the 96-wells (Additional file 1: Figure S4e).

### Immunofluorescence and cryosectioning

Cells were fixed with 4% PFA, permeabilized with 0.2% Triton X-100 at room temperature, and then blocked in 3% goat serum, followed by incubation with primary (TBR1 - Abcam, ab31940; CTIP2 – Abcam, ab18465; β-3 tubulin – Biolegend, 801,202; PSD-95 – Synaptic Systems, 124,011; Synaptophysin 1 – Synaptic Systems, 101,002; Transthyretin – Novus Biologicals, NBP2–52575, Prostaglandin D Synthase (PTGDS) – Abcam, ab182141) and secondary antibodies (Alexa Flours, Invitrogen). The nuclei were counter-stained with 49,6-diamidino-2-phenylindole (DAPI, Sigma). Cryosectioning of neurospheres was performed as previously described [61]. Cells were imaged using an Observer D1 (Zeiss) microscope or Olympus SD-OSR spinning-disk confocal microscope (60x oil immersion). The images were processed using Zen 2 (Zeiss), MetaMorph, or Image J (brightness and contrast adjustments, thresholding for composite images) and assembled using Adobe Photoshop CS6.

Antibody validation: TBR1: validated on Mouse Hippocampus Tissue Lysate, Rat Hippocampus Tissue Lysate, Human cerebral cortex. CTIP2: validated by IHC on adult mouse hippocampus and adult mouse spinal cord and by ICC on neonatal mouse hippocampal cultured neurons. b3-tubulin: Quality control tested by formalin-fixed paraffin-embedded immunohistochemical staining. PSD-95: Knock-out verified, validated by IF on rat hippocampal neurons. Synaptophysin I: Does not cross-react with other synaptophysins, validated by IF on hippocampal neurons. TTR: Validated by IF analysis of A549 and MCF-7 cells and IHC of human liver tissue. PTGDS: Validated by IF on HEPG2 cells and IHC on human prostate tissue. All information is from supplier product data sheets.

### Calcium imaging

The intracellular Ca^2+^ oscillations in human cortical neuron and rat glia co-cultures were assessed using the FLIPR Calcium 6 Kit (Molecular Devices LLC, San Jose, California). Briefly, 96-well Greiner μ-clear plates (655097) were seeded with 2500 rat glia (Lonza, R-CXAS-520) per well in Ph IV media and cultured for 7 days. Human cortical neurospheres were dissociated with papain as described above at DIV 56, and 50,000 single cells per well were plated on rat glia in phase IV media. Co-cultures were maintained for 4 weeks with twice-weekly 50% medium exchange. Cells were loaded with calcium 6 dye for an hour which was reconstituted in imaging buffer (NaCl 2.5 mM, KCl 125 mM, KH_2_PO_4_ 1.25 mM, CaCl_2_ 2 mM, MgCl_2_ 2 mM, HEPES (acid) 25 mM, D-glucose 30 mM, pH 7.4, filter-sterilized). Kinetics of Ca^2+^ oscillations were determined as fluorescence intensity at 540 nm following excitation at 480 using the FDSS 7000EX Functional Drug Screening System (Hamamatsu) maintained at a constant 37 °C throughout the assay. A total of 3000 reads per assay were recorded. The exposure time per read was 100 ms with sensitivity set to 1.

### Single-cell RNA-sequencing of neuronal cells

Cells were resuspended to 1 million cells/mL and run through the 10X Chromium, Version 2, single-cell RNA-seq pipeline per vendor’s instructions. Reverse transcription master mix was prepared from 50 μL RT reagent mix (10X, 220,089), 3.8 μL RT primer (10X, 310,354), 2.4 μL additive A (10X, 220,074), and 10 μL RT enzyme mix (10X, 220,079). 4.3 μL cell solution was mixed with 29.5 μL H_2_O and 66.2 μL reverse transcription master mix. Ninety-microliter sample was loaded onto the 10X Single Cell 3′ Chip along with 40 μL barcoded gel beads and 270 μL partitioning oil, and the microfluidics system was run to match gel beads with individual cells. The droplet solution was then slowly transferred to an 8-tube strip, which was immediately incubated for 45 min at 53 °C to perform reverse transcription, then 5 min at 85 °C. The sample was treated with 125 μL recovery agent (10X, 220,016), which was then removed along with the partitioning oil. Two hundred microliters of cleanup solution containing 4 μL DynaBeads MyOne Silane Beads (Thermo Fisher, 37002D), 9 μL water, 182 μL Buffer Sample Clean Up 1 (10X, 220,020), and Additive A (10X, 220,074) was added to the sample, and the solution was mixed 5 times by pipetting and allowed to incubate at room temperature for 10 min. Beads were separated via magnetic separator and supernatant was removed. While still on the magnetic separator, the beads were then washed twice with 80% ethanol. The separator was then removed and the beads were resuspended in 35.5 μL elution solution consisting of 98 μL Buffer EB (Qiagen, 19,086), 1 μL 10% Tween 20 (Bio-Rad, 1,610,781), and 1 μL Additive A (10X, 220,074). The solution was then incubated for 1 min at room temperature and placed back onto the magnetic separator. Thirty-five microliters of eluted sample was transferred to a new tube strip. cDNA amplification reaction mix was prepared from 8 μL water, 50 μL Amplification Master Mix (10X, 220,125), 5 μL cDNA Additive (10X, 220,067), and 2 μL cDNA Primer Mix (10X, 220,106). Sixty-five microliters of amplification master mix was added to the sample, mixed 15 times via pipetting, and briefly centrifuged. The sample then underwent 12 amplification cycles (15 s at 98 °C, 20 s at 67 °C, 1 min at 72 °C).

SPRIselect beads (Beckman Coulter, B23318) were then applied at 0.6X, and solution was mixed 15 times via pipetting. The sample was incubated at room temperature for 5 min, placed onto a magnetic separator, and washed twice with 80% ethanol. Sample was air-dried for 2 min and eluted in 40.5 μL Buffer EB. cDNA yield was measured on a 2100 Bioanalyzer (Agilent, G2943CA) via DNA High Sensitivity Chip (Agilent, 5067–4626).

Fragmentation mix was prepared at 4 °C from 10 μL fragmentation enzyme blend (10X, 220,107) and 5 μL fragmentation buffer (10X, 220,108). Thirty-five microliters of sample cDNA was then added to the chilled fragmentation mix. Sample was incubated for 5 min at 32 °C, then 30 min at 65 °C to conduct enzymatic fragmentation, end repair, and A-tailing. Sample was then purified using 0.6X SPRIselect reagent (see above). Adaptor ligation mix was prepared from 17.5 μL water, 20 μL ligation buffer (10X, 220,109), 10 μL DNA ligase (10X, 220,110), and 2.5 μL Adaptor Mix (10X, 220,026). The ligation mix was added to 50 μL of sample and mixed 15 times via pipetting. Sample was then incubated for 15 min at 20 °C to conduct the ligation. The sample was purified using 0.8X SPRIselect reagent (see above). Sample index PCR mix was prepared from 8 μL water, 50 μL Amplification Master Mix (10X, 220,125), and 2 μL SI-PCR Primer (10X, 220,111). 60 μL sample index PCR mix, 30 μL purified sample, and 10 μL of sample index (10X, 220,103) were combined and mixed 15 times via pipetting. Indexing was conducted via 9 cycles of 20 s at 98 °C, 30 s at 54 °C, then 20 s at 72 °C. Sample was purified via double-sided SPRI selection at 0.6X and 0.8X, respectively. Sample was then quantified via DNA High Sensitivity Chip.

Additional quantification was conducted via KAPA Library Quantification Kit (Illumina, KK4828–07960166001). Sample was diluted at 10-fold increments from 1:100 to 1:1,000,000, and mixed 1:9 with KAPA qPCR mix. qPCR was conducted on a Viia7 qPCR machine (Life Technologies).

Sample was then sequenced on a HiSeq 4000 (Illumina) using 2 × 50-cycle SBS kits (Illumina, FC-410-1001). Sample library was diluted to 2 nM in EB buffer with 1% PhiX spike-in. Five microliters nondenatured library was then mixed with 5 μL 0.1 N NaOH, then vortexed and briefly centrifuged. Denaturing was conducted at room temperature for exactly 8 min, then stopped via the addition of 5 μL 200 mM Tris-HCl pH 8.0 (Fluka, 93,283). Sample was mixed, briefly centrifuged, and placed on ice. ExAmp reaction mix (Illumina, PE-410-1001) was prepared, added to the sample, and clustering was done on a HiSeq 4000 flow cell via cBot2 (Illumina). The library was then sequenced with paired-end reagents, with 26xRead 1 cycles, 8xi7 index cycles, and 98xRead 2 cycles.

The 10X Cell Ranger 1.3.1 pipeline was utilized to convert raw BCL files to cell-gene matrices. FASTQ files were aligned to the GRCh37.75 human reference genome, UMI-filtered, and barcodes were matched via the CellRanger count script.

### Computational analysis

#### Software requirements and scRNA-seq workflow

All computational analysis was carried out using R v. 3.4.1 with Bioconductor v. 3.5. We assembled a modular workflow for the analysis of scRNA-seq data that contains five modules: (i) quality control, (ii) data normalization, (iii) feature selection, (iv) clustering, and (v) identification of marker genes (Fig. 2a). Based on recent publications, the quality control and normalization modules were based on the popular scater [29] and scran [62] packages. Scran was set as the default normalization based on a recent benchmarking study by Vallejos et al. [63] showing that scran was superior for recovering true size factors compared to other methods. For the marker gene identification module we used the Wilcoxon test [64] by default and provided wrappers to MAST [21] and Limma-trend [65], based on Soneson et al.*’*s [66] comprehensive assessment of a large number of DE analysis methods for their performance for controlling type I and type II error rates while being scalable to large datasets.

#### Generation of synthetic data

A synthetic dataset was generated based on estimated parameters for the gene-wise mean *μ*_*i*_ and variance ${\sigma}_i^2$ from experimentally determined counts of 1000 K562 cells from our benchmarking dataset.

Because gene expression within each cell is typically not independent but cells that have high/low count number for one gene also tend to have high/low counts for another, we sampled for each cell *j* a scaling factor *θ*_*j*_ such that ${\log}_2\left({\theta}_j\right)\sim \mathcal{N}\left(\mathrm{0,0.25}\right)$, as described in [62]. Simulated counts for gene *i* and cell *j* were generated by sampling from a negative binomial with mean

$$ {\mu}_{ij}={\theta}_j\ast {\mu}_i $$

and dispersion^1^

$$ {\lambda}_{ij}=\frac{\mu_{ij}^2}{\ {\sigma}_i^2-{\mu}_{ij}} $$

A second-order polynomial was fit to the sample variance as a function of the mean in logarithmic space as described in [8]. This polynomial served as an estimate of the global mean-variance relationship. Replacing the term ${\sigma}_i^2$ in the equation above with this estimate, the dispersion can be expressed as a function of *μ*_*ij*_:

$$ {\lambda}_{ij}=\frac{\mu_{ij}^2}{f\left({\mu}_{ij}\right)-{\mu}_{ij}} $$

where

$$ f\left({\mu}_{ij}\right)=2\hat{\mkern6mu} \left(a\ast \mathrm{lo}{\mathrm{g}}_2\left({\mu}_{ij}\right)\hat{\mkern6mu} 2+b\ast {\log}_2\left({\mu}_{ij}\right)+c\right) $$

is derived from the second-order polynomial approximating the gene-wise variance as a function of mean expression. For genes exhibiting Poissonian behavior (i.e., equal mean and variance), we set *λ* to a fixed value of 10^10^.

Main cell populations were obtained by permutation of the expression values of 100 randomly chosen genes with mean counts larger than 2.

Cell subgroups characterized by high expression of a small set of marker genes were generated by replacing the base mean values *μ*_*i*_ in a small set of genes with low expression (*μ*_*i*_ < 0.1) by a value of 2^*x*^ where $x\sim \mathcal{N}\left(\mathrm{2.5,1}\right)$. Thus, the upregulated genes exhibit a log2 fold change of 2.5 on average.

### Simulating varying degrees of subtlety in transcriptional differences

An initial small dataset was subsampled from the benchmarking (8 human cell lines) dataset, comprising 100 HEK293, 125 Ramos, and between 10 Jurkat cells. We used scran to predict cell cycle stage and only included cells in G1 phase.

From this initial dataset, 25 Ramos cells were held out. From the remaining dataset (100 HEK293, 100 Ramos, 10 Jurkat), datasets with varying incidence of a rare cell type and subtlety (i.e., degree of difference to closest neighbor) of its transcriptional signature were generated in silico, following an approach recently described by Crow et al. [39]: First, a number of Jurkat cells (i.e., incidence of 2, 5, or 10) were sampled from the initial dataset. Then, to simulate varying degrees of transcriptional difference between the rare cell type (Jurkat) and its closest abundant cell type (Ramos), an increasing fraction of gene expression values, ranging from 0 to 0.995 in steps of 0.05 (0.045 for the very last step) in the Jurkat cells were replaced by the respective values in the held out Ramos cells. This fraction of replaced expression values is referred to as subtlety.

This procedure was repeated 5 times for each incidence of the rare cell type and each value of the subtlety parameter.

The performance of CellSIUS, GiniClust2, and RaceID3 was evaluated in terms of recall, precision and true negative rate (TNR) for each configuration. To this end, a confusion matrix between the true cell type and the predicted cell type was generated. “Main clusters” were defined as the two clusters containing the majority of the HEK293 and Ramos cells, respectively. The TPR was then defined as the fraction of Jurkat cells that were not assigned to the main clusters, precision was defined as the fraction of Jurkat cells among all cells not assigned to the two main clusters, and the TNR was defined as the fraction of HEK293 and Ramos cells that were assigned to the main clusters.

### Data pre-processing

Initial pre-processing was applied to each batch of cell lines separately prior to annotating cell types.

First, cells were filtered based on the total number of detected genes, total UMI counts, and the percentage of total UMI counts attributed to mitochondrial genes. Cutoffs were set individually per batch based on the overall distributions (Additional file 1: Table S5).

Second, genes have to present with at least 3 UMIs in at least one cell. After this initial QC, remaining outlier cells were identified and removed using the *plotPCA* function from the scater [29] R package with *detect_outliers* set to TRUE.

Data were normalized using scran [62], including a first clustering step as implemented in the *quickCluster* function and with all parameters set to their default values.

### Cell type annotation

First, the top 10% overdispersed genes were selected using the NBDrop method described in [28]. Cell types were then annotated based on Pearson’s correlation of the expression profile (log_2_(normalized counts+ 1)) of the selected features with bulk RNA-seq data obtained for each individual cell line (Fig. 1a, b). For the batches 1–3 that contained only two cell lines each, the Pearson’s correlation coefficients were scaled to *z*-scores prior to the assignment, and for batch 4, the raw correlation values were used instead. A cell was then assigned to the cell line with the highest value unless this maximum was below 0.2 or if the second highest value was within 5% of the maximum in which case no assignment was given. We found that the latter applied only to a small percentage of cells (1–2%), which most likely correspond to cell doublets. Furthermore, for the cell line mixes, IMR90/HCT116 and A549/Ramos additional potential doublets were identified and excluded from the cell line assignment employing a visual inspection of the tSNE plot by looking for (small) clusters of cells having high correlation to both cell lines as well as a high UMI count (Additional file 1: Table S3).

After cell type annotation, the raw count matrices from all four batches were concatenated. Cells that had not passed the initial QC or could not be annotated were discarded. The gene filtering step described above was then repeated for the aggregated dataset, leaving a final cleaned dataset containing a total of 12,718 genes and 11,678 cells.

### Dimensionality reduction and calculation of distance matrix

The original expression (log2(normalized counts + 1) coordinates were projected into low-dimensional space by PCA, using an implicitly restarted Lanczos method as implemented in the irlba [36] R package. The number of dimensions to retain was determined by visual inspection of a scree plot. It was 10 for all cell line data and 12 for the neuron dataset, and the first *k* principal components accounted for 40–50% of the total variance in each case. Cell-cell distances (Euclidean or Pearson, Additional file 1: Table S2) were then calculated on these projections.

### Benchmarking of clustering approaches

The accuracy of each prediction was assessed by the adjusted rand index (ARI). Given two partitions *X* = *X*_1_, … , *X*_*m*_ and *Y* = *Y*_1_, … , *Y*_*k*_ of a set S with *n* elements, the ARI is defined as:

$$ \mathrm{ARI}=\frac{\sum_{ij}\left(\genfrac{}{}{0pt}{}{n_{ij}}{2}\right)-\left[{\sum}_i\left(\genfrac{}{}{0pt}{}{a_i}{2}\right){\sum}_j\left(\genfrac{}{}{0pt}{}{b_j}{2}\right)\right]/\left(\genfrac{}{}{0pt}{}{n}{2}\right)\kern0.75em }{\frac{1}{2}\left[{\sum}_i\left(\genfrac{}{}{0pt}{}{a_i}{2}\right)+{\sum}_j\left(\genfrac{}{}{0pt}{}{b_j}{2}\right)\right]-\left[{\sum}_i\left(\genfrac{}{}{0pt}{}{a_i}{2}\right){\sum}_j\left(\genfrac{}{}{0pt}{}{b_j}{2}\right)\right]/\left(\genfrac{}{}{0pt}{}{n}{2}\right)\kern0.75em } $$

where *n*_*ij*_ denotes the elements that are common between *X*_*i*_ and *Y*_*j*_, and *a*_*i*_, *b*_*j*_ are the total number of elements in *X*_*i*_ and *Y*_*j*_, respectively.

### CellSIUS

CellSIUS detects cell subpopulations and their gene signatures (Fig. 3a). Starting from an initial partitioning of *N* cells into *m* clusters *C*_1_, … , *C*_*m*_, the method identifies cell subpopulations and their signatures as follows:

1. Identification of genes with bimodal expression: For each gene *g*_*i*_, within each cluster *C*_*j*_, a one-dimensional *k*-means clustering is used to partition the cellular expression levels (log2 normalized UMI counts) into two groups (“low” and “high”). Candidate marker genes are selected according to three criteria: (i) the average expression fold change between “low” and “high” is at least 2 on a log2-scale, (ii) less than a user defined percentage (50% by default) of all cells in cluster *C*_*j*_ fall in the “high” category, and (iii) there is a significant difference (*t* test and Benjamini-Hochberg correction, *p* value < 0.1) between the “low” and “high” expression values.
2. Testing cluster specificity: For the list of candidate genes, it is assessed whether the cell subgroup expressing them is specific to cluster *C*_*j*_. Required for each gene *g*_*i*_ are (i) a significant difference in the expression of *g*_*i*_ in cells with “high” expression compared to cells not in Cj (*t* test and FDR correction, *p* value < 0.1) and (ii) the average expression fold change between all cells with “high” expression and all other cells with non-zero expression of *g*_*i*_ to be at least 1 on a log2-scale.
3. Identification of correlated gene sets: For each cluster *C*_*j*_, the correlation matrix of the expression of all candidate genes *g*_1, . . , *n*_ across all cells in cluster *C*_*j*_ is transformed into a graph where genes correspond to nodes and edges are weighted by correlations between them. Edges with weights below a fixed threshold are assigned a weight of 0. By default, this threshold is set to the 95th percentile of all correlations if this value lies between 0.35 and 0.5, and to the lower and upper bound if it is below or above, respectively. The lower bound is set such that it is higher than the maximum of all gene-wise correlations on simulated data from an entirely homogeneous population, which serves as an estimate of the background correlation. Setting an upper bound ensures that gene sets are not falsely split in cases where all candidate genes are highly correlated. Subsequently, MCL [33, 34] is used to identify correlated gene sets, denoted *s*_*jk*_, where *j* is the index of the main cluster and *k* the index of the gene set within this cluster.
4. Assigning cells to subgroups: For each cluster *C*_*j*_ and each gene set *s*_*jk*_, a one-dimensional *k*-means is run on the mean expression of *s*_*jk*_. Cells falling in the “high” mode of this clustering are assigned to a new cluster *C*_*jk*_.
5. Final cluster assignment: Cells are assigned to a final cluster which is the combination of all subgroups they belong to. This means if a cell belongs to two subgroups A and B, it will be assigned to a new subgroup AB. The gene signatures for this new subgroup correspond to the union of gene signatures A and B. Only subgroups characterized by a minimum of min_n_genes (default: 3 genes) are considered.

### Identification of rare cell types with RaceID and Giniclust

RaceID3 [38] was obtained from GitHub (dgrun/RaceID3_StemID2, version as of March 26th 2018). Analysis was run with all parameters at their default values, except that we fixed the initial clusters (RaceID@kpart) instead of determining them by *k*-medoids. On biological data (cell line subset 2 and neuronal population), we in addition changed the probability threshold to 10^−20^ and set the minimum number of outlier genes (outlg) to 3. This adjustment was made because the default cutoffs in RaceID are not very stringent and resulted in extensive overclustering of the data.

GiniClust2 [19] was obtained from GitHub (dtsoucas/GiniClust2, version as of 4 May 2018). All analysis was run with dataset-specific parameters: MinPts = 3, eps = 0.45, *k* = 2 for the simulated data, and MinPts = 3, eps = 0.45, *k* = 8 for the cell line dataset. All other parameters were set to their defaults.

### Trajectory analysis using monocle

Analysis was run using monocle version 2.4.0. As input, the counts of the top 10% genes selected by NBDrop were used. Prior to monocle analysis, all genes annotated with the GO term cell cycle (GO:0007049) as well as mitochondrial genes and genes encoding ribosomal proteins were removed from the dataset. All parameters were set to default values.

## Additional files

Additional file 1: **Figure S1.** tSNE visualization of potential confounders in cell line dataset. **Figure S2.** Generation of synthetic scRNA-seq data. **Figure S3.** Parameter sensitivity analysis of CellSIUS. **Figure S4.** In vitro differentiation of hPSCs into cortical excitatory neurons. **Figure S5.** hPSC-derived cortical neurons express characteristic marker genes. **Figure S6.** Identification of cell subgroups in neuronal populations. **Figure S7.** Comparison of neuronal population markers to scRNA-seq data from the developing human cortex. **Table S1.** Composition of full and subsampled cell line datasets. **Table S2.** Overview of clustering algorithms benchmarked in this study. **Table S3.** Medium composition for the in vitro differentiation of cortical excitatory neurons from human pluripotent stem cells in suspension. **Table S5.** Sequencing statistics and QC cutoffs per batch. (PDF 3452 kb)

Additional file 2: **Table S4.** DE analysis between subclusters and main clusters in the neuroscience dataset. The file contains one sheet per comparison. All sheets are listed below: G.sub_1_vs_all_G: compares the G.sub_1 population to all other cells in the G cluster. G.sub_2_vs_all_G: compares the G.sub_2 population to all other cells in the G cluster. G.sub_3_vs_all_G: compares the G.sub_3 population to all other cells in the G cluster. CR.sub_vs_all_CR: compares the CR.sub population to all other cells in the CR cluster. NP.sub_vs_all_NP: compares the NP.sub population to all other cells in the NP cluster. N.sub_1_vs_all_N: compares the N.sub_1 population to all other cells in the N cluster. N.sub_2_vs_all_N: compares the N.sub_2 population to all other cells in the N cluster. Each sheet contains the following columns: Gene_id: Ensembl gene ID. Mean_exprs: Mean expression [log2(normalized counts + 1)] across the whole dataset. Mean_in_subgroup: Mean expression in the respective subgroup. Pval, adj_pval: *p* value (Wilcoxon test), adj_pval is adjusted *p* value (Benjamini-Hochberg). Log2fc: Fold change, calculated as the difference in mean[log2(normalized counts + 1)]. DE_flag: is TRUE if abs(log2fc) > 0.5 and adj_pval < 0.05. Chr, symbol, eg, gene_biotype, description: Additional gene info (chromosome, gene symbol, entrez gene identifier, gene biotype, short description of gene function). (XLSX 8049 kb)

Additional file 3: Review history (DOCX 58 kb)

## Data Availability

ScRNA-seq data of human cell lines have been deposited in the NCBI Short Read Archive (SRA) under accession number SRA: PRJNA484547 [69]. ScRNA-seq data of differentiation of cortical excitatory neurons from human pluripotent stem cells in suspension have been deposited in the NCBI Short Read Archive (SRA) under accession number SRA: PRJNA545246 [70]. The workflow written in the R programming language is deposited in GitHub (https://github.com/Novartis/scRNAseq_workflow_benchmark) and Zenodo (DOI: 10.5281/zenodo.3237742) [71]. The code, vignette, and an example dataset for the computational workflow are included in the repository. The CellSIUS is deposited in GitHub (https://github.com/Novartis/CellSIUS) [72] and Zenodo (DOI: 10.5281/zenodo.3237749) [73] as a standalone R package. It requires *R* ≥ 3.4.1 and uses an external installation of the Markov Clustering Algorithm (MCL) [33, 34]. The R implementation is platform independent; the external MCL runs on any UNIX platform. The codes and processed data to reproduce the analyses presented here are uploaded in Zenodo (10.5281/zenodo.3238275) [74]. All the open source released repositories are under the “Apache License 2.0”.

## Abbreviations

ARI: Adjusted Rand index
CP: Choroid plexus
CR: Cajal-Retzius
CSF: Cerebrospinal fluid
DANB: Depth-adjusted negative binomial
DE: Differential expression
G: Glia
GC: Glycolytic cell
GMM: Gaussian mixture model
hPSC: Human pluripotent stem cell
HVG: High variance gene
IP: Intermediate progenitor
N: Neuron
NP: Neocortical progenitor
oRG: Outer radial glia
PCA: Principal component analysis
scRNA-seq: Single-cell RNA sequencing

## Footnotes

1 We use this nomenclature in order to be consistent with the definition in R. Note that there is an alternative nomenclature, which defines *α* = 1/*λ* as dispersion and is used in edgeR [67] and DESeq2 [68].
