# Supplementary material for: CellSIUS provides sensitive and specific detection of rare cell populations from complex single-cell RNA-seq data
Source: Genome Biol. 2019 Jul 17;20:142. doi: 10.1186/s13059-019-1739-7 (PMC6637521; doi:10.1186/s13059-019-1739-7)
Supplement: Supplementary file 1 — Figure S1. tSNE visualization of potential confounders in cell line dataset. Figure S2. Generation of synthetic scRNA-seq data. Figure S3. Parameter sensitivity analysis of CellSIUS. Figure S4. In vitro differentiation of hPSCs into cortical excitatory neurons. Figure S5. hPSC-derived cortical neurons express characteristic marker genes. Figure S6. Identification of cell subgroups in neuronal populations. Figure S7. Comparison of neuronal population markers to scRNA-seq data from the developing human cortex. Table S1. Composition of full and subsampled cell line datasets. Table S2. Overview of clustering algorithms benchmarked in this study. Table S3. Medium composition for the in vitro differentiation of cortical excitatory neurons from human pluripotent stem cells in suspension. Table S5. Sequencing statistics and QC cutoffs per batch. (PDF 3452 kb) [file 13059_2019_1739_MOESM1_ESM.pdf]

# Additional file 1: Supplementary figures and tables

This file contains supplementary figures 1-7 and supplementary tables 1-3 and 5. Supplementary table 4 is provided as a separate file.

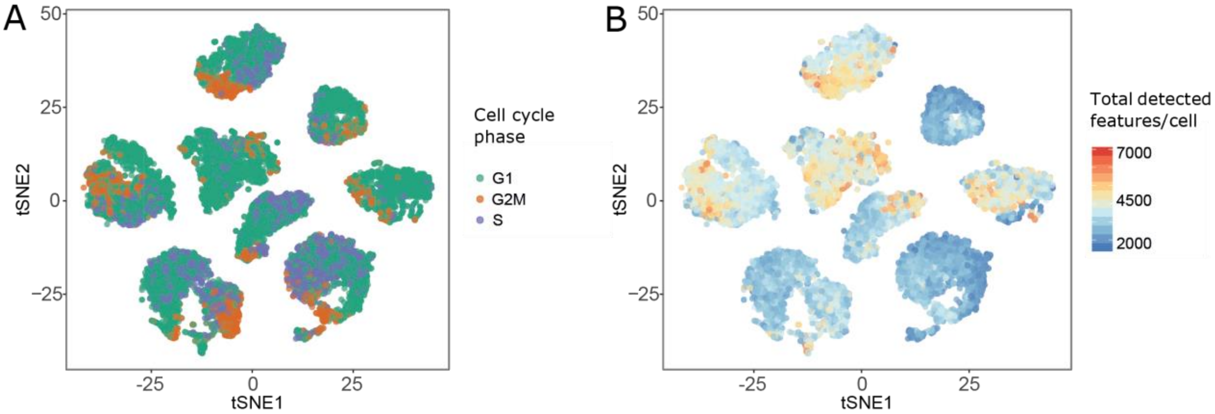


**Figure S1:** tSNE visualization of potential confounders in cell line dataset. A: tSNE-map, colored by predicted cell cycle phase. B: tSNE-map, colored by total detected features per cell.

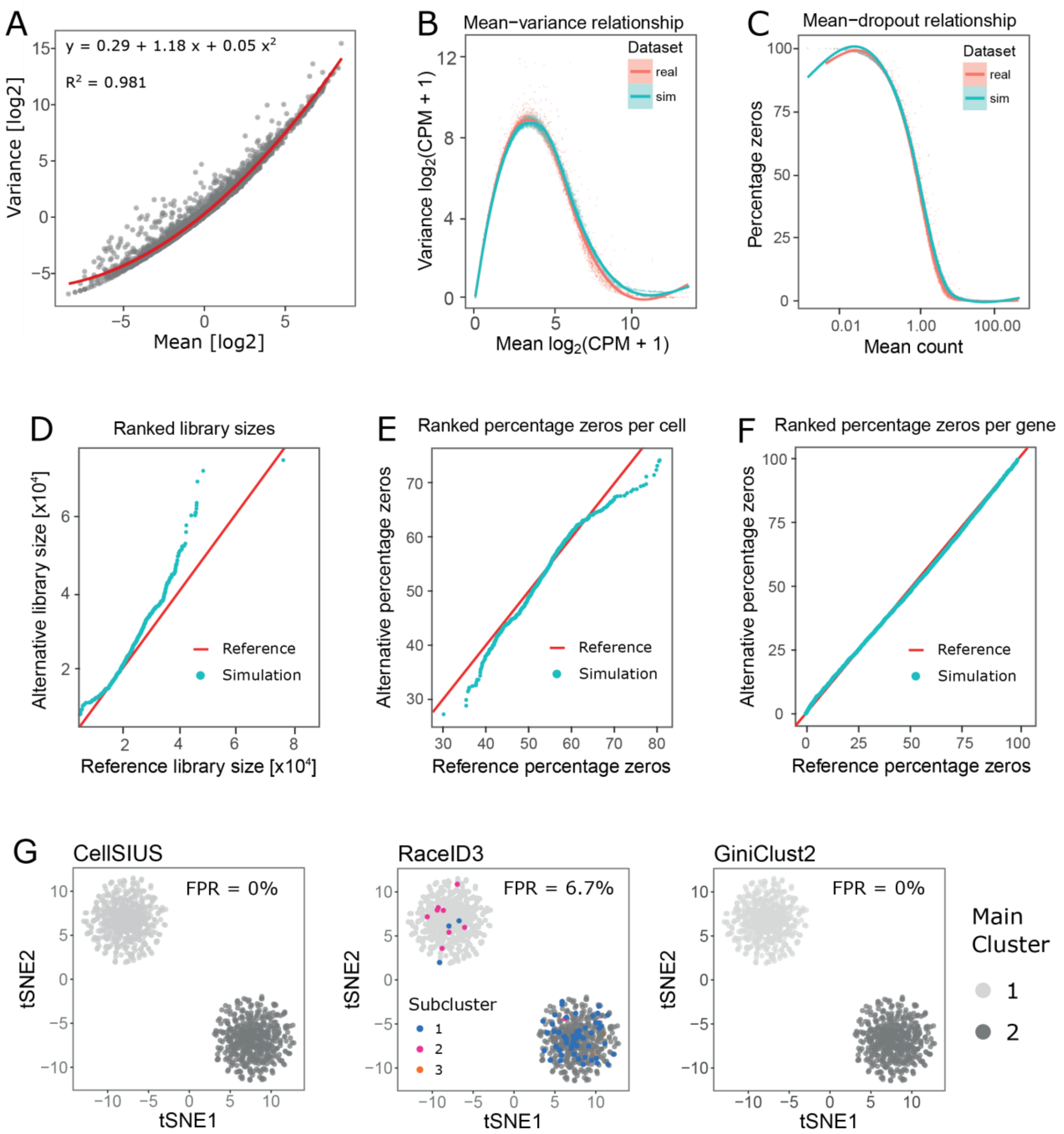


**Figure S2:** Generation of synthetic scRNA-seq data. A: Gene-wise mean-variance trend. The red line indicates a second order polynomial fit to the data that was used to estimate the expected variance as a function of the mean. B-F: Synthetic data (cyan) recapitulate the properties of experimental data (red). Plots show the relationship between the gene-wise mean and variance (B), the relationship between the mean count per gene and the fraction of zero counts / dropouts (C), ranked total number of counts per cell (D), ranked percentage of zero counts per cell (E) and ranked percentage of zeros per gene (F). G: Estimating false positive rates (FPR). The data shown are from two entirely homogeneous populations. CellSIUS and GiniClust do not falsely report rare cell types, whereas RaceID does.

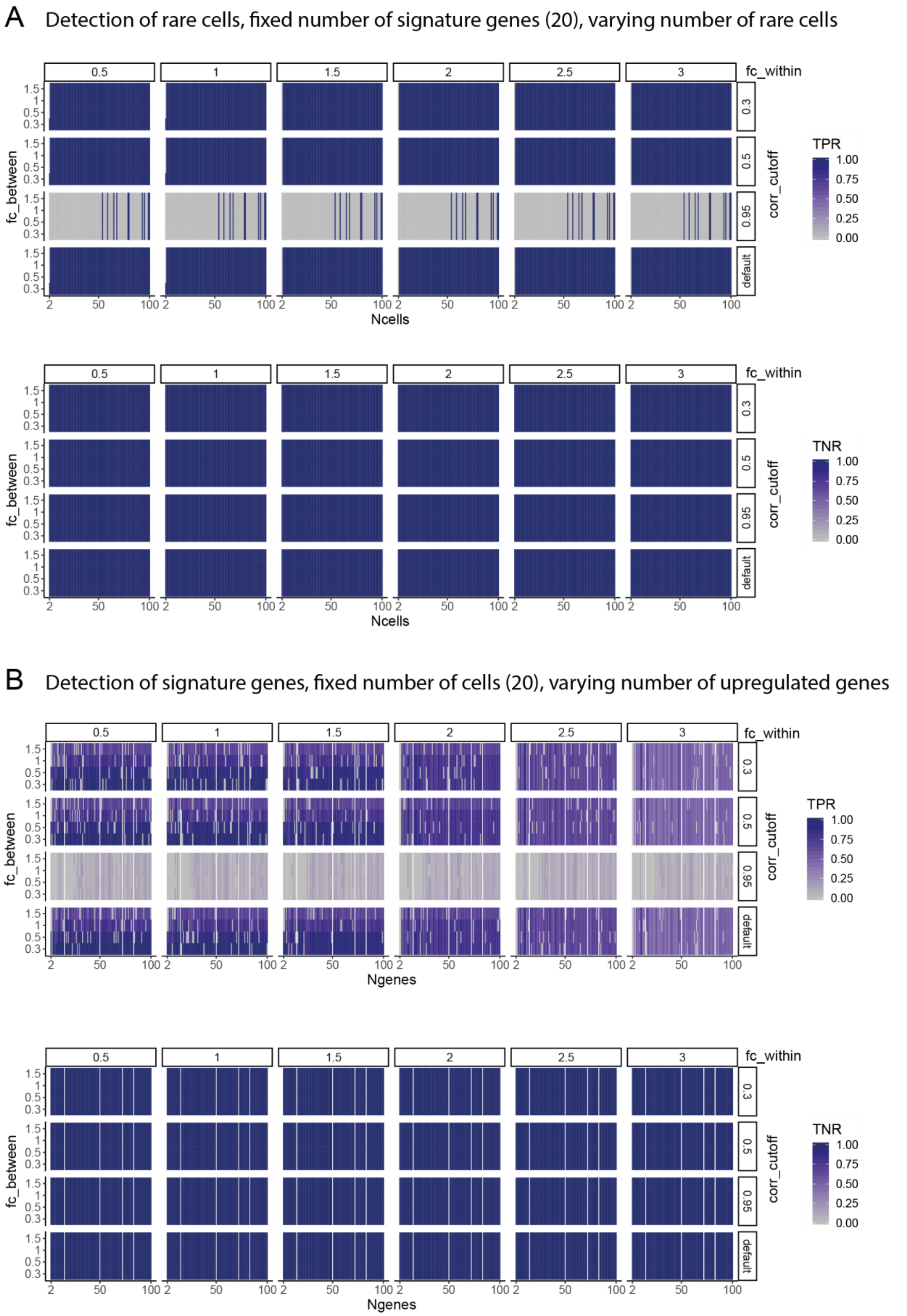


**Figure S3:** Parameter sensitivity analysis of CellSIUS. The heatmaps show the true positive rate (TPR) and true negative rate (TNR) with respect to (A) detection of rare cells and (B) detection of gene signatures for different values of the key parameters fc_between (y-axis), fc_within (panel columns) and corr_cutoff (panel rows).

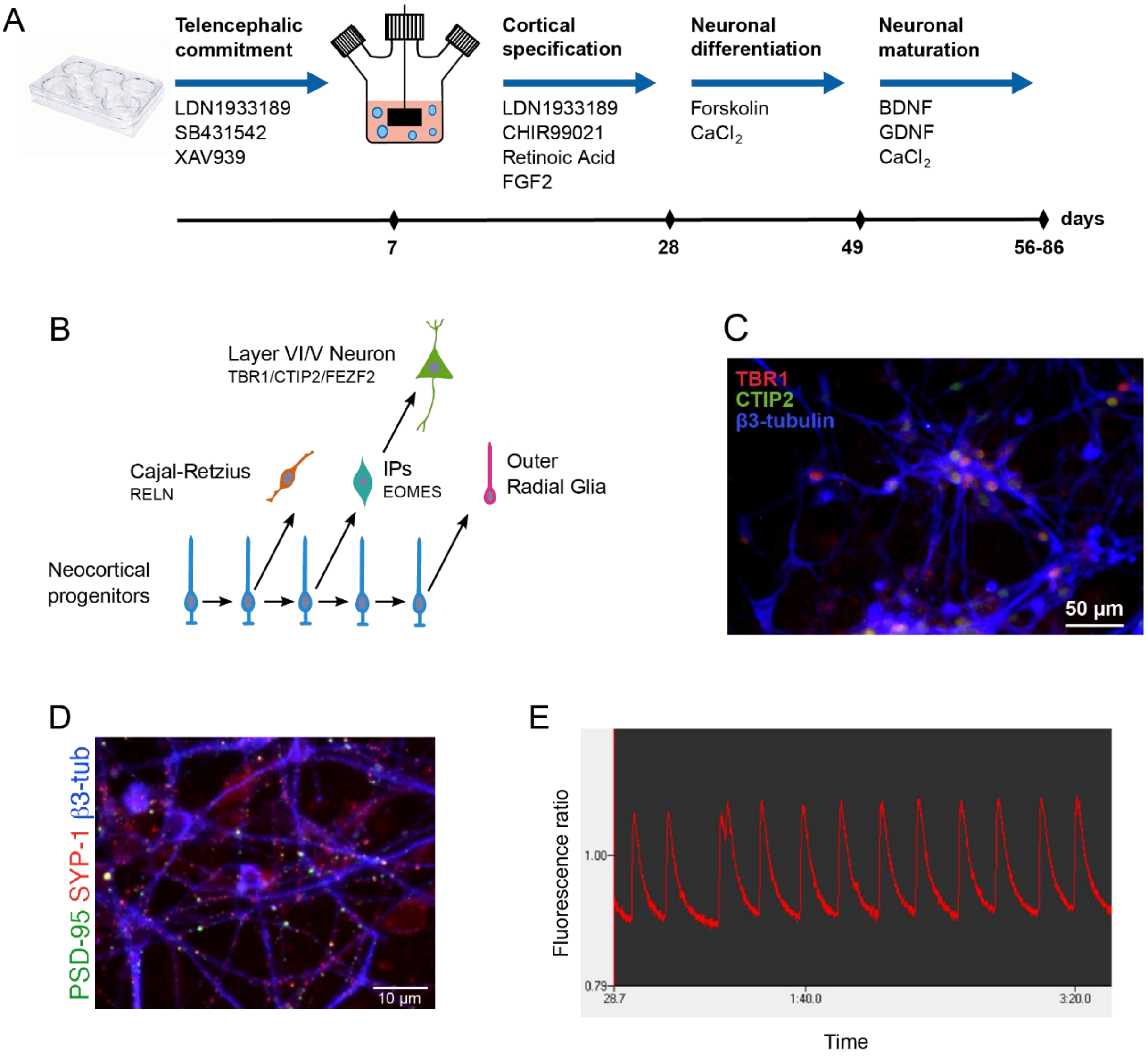


**Figure S4:** In vitro differentiation of hPSCs into cortical excitatory neurons. A: Schematic overview of the 3D cortical spheroid differentiation protocol. hPSCs grown as a monolayer were patterned to telencephalon and differentiated in suspension culture by stage-specific application of small molecules. B: Illustration of neurogenesis. After committing to definitive neuroepithelia and restricting to dorsal telencephalic identity, hPSCs generate neocortical progenitors which further give rise to Cajal-Retzius (CR) cells, EOMES+ intermediate progenitors (IPs), layer VI and V cortical excitatory neurons (N) and outer radial glia (oRG). C: Immunofluorescence confirms the robust expression of deep-layer cortical neuronal markers (TBR1, CTIP2) in hPSC derived neurons (β-3-tubulin). D: Immunohistochemical staining against post-synaptic density protein (PSD-95), Synaptophysin I (SYP-1) and β3-tubulin. E:  Representative spontaneous calcium oscillation signal trace from a well of a high-density human cortical neuron / rat glia co-culture in 96-well format.  Cells were loaded with the fluorescence calcium indicator FLIPR Calcium 6 dye (Molecular Probes) and observed for their spontaneous behaviour using the fluorescence plate reader FDSS7000EX (Hamamatsu).

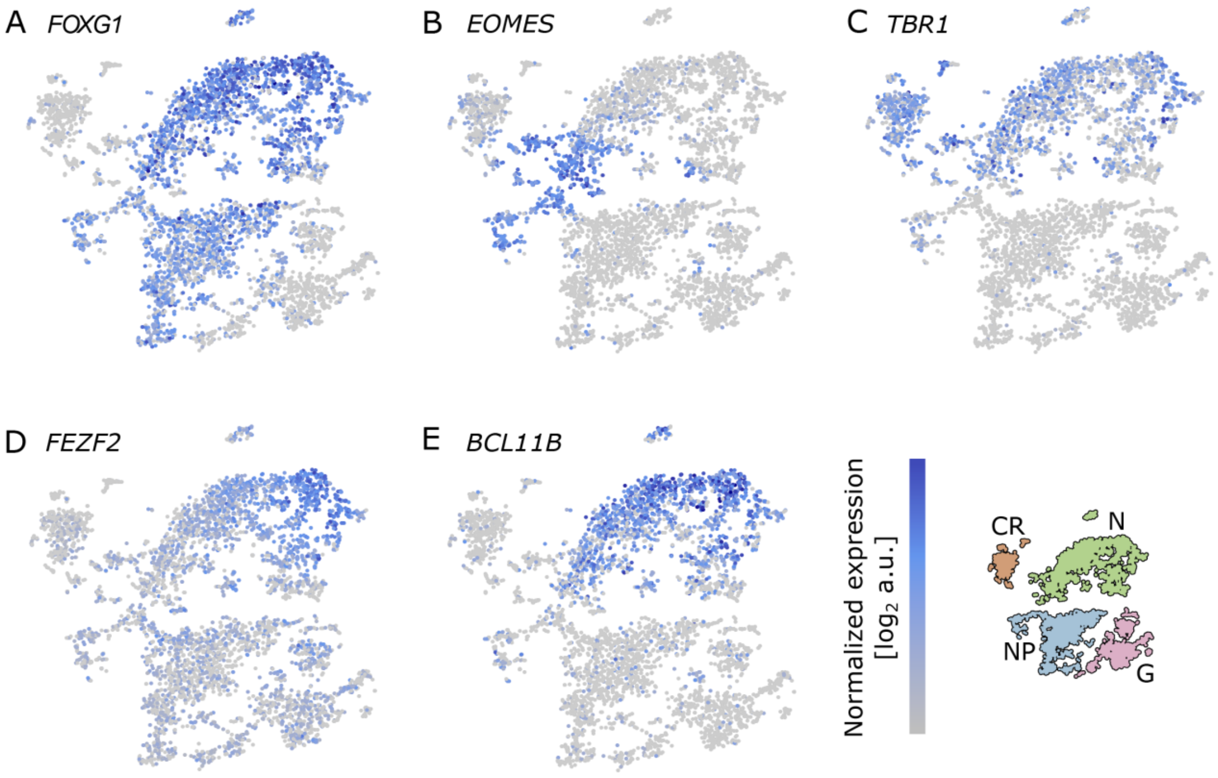


**Figure S5:** hPSC derived cortical neurons express characteristic marker genes. Shown are tSNE projections, colored by expression of A: FOXG1, B: EOMES, C: TBR1, D: FEZF2, E: BCL11B. The small map on the right shows the location of the main population on the tSNE projection (N: Neuron, NP: neuroepithelial progenitor, G: mixed glial cells, CR: Cajal-Retzius cells).

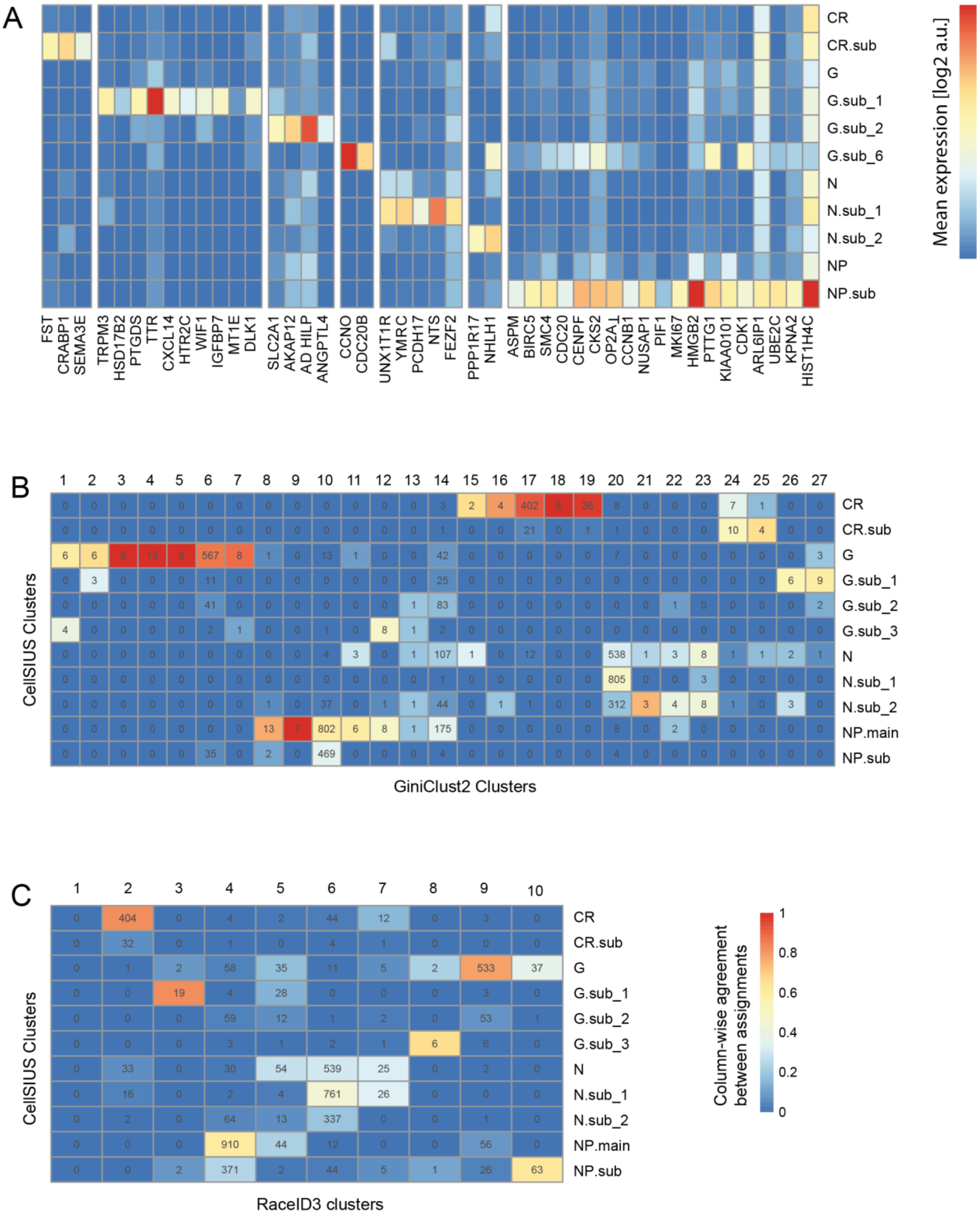


**Figure S6:** Identification of cell subgroups in neuronal populations. A: Subgroups and their markers identified by CellSIUS. Rows correspond to clusters, columns to genes. Colors indicate mean expression level per cluster. B,C: Confusion matrix between cluster assignments by CellSIUS and Giniclust2 (B) and CellSIUS and RaceID3 (C). Rows correspond to CellSIUS assignment, columns to assignment by Giniclust2 and RaceID3, respectively. Numbers indicate the number of cells, colors indicate the degree of agreement, calculated per column.

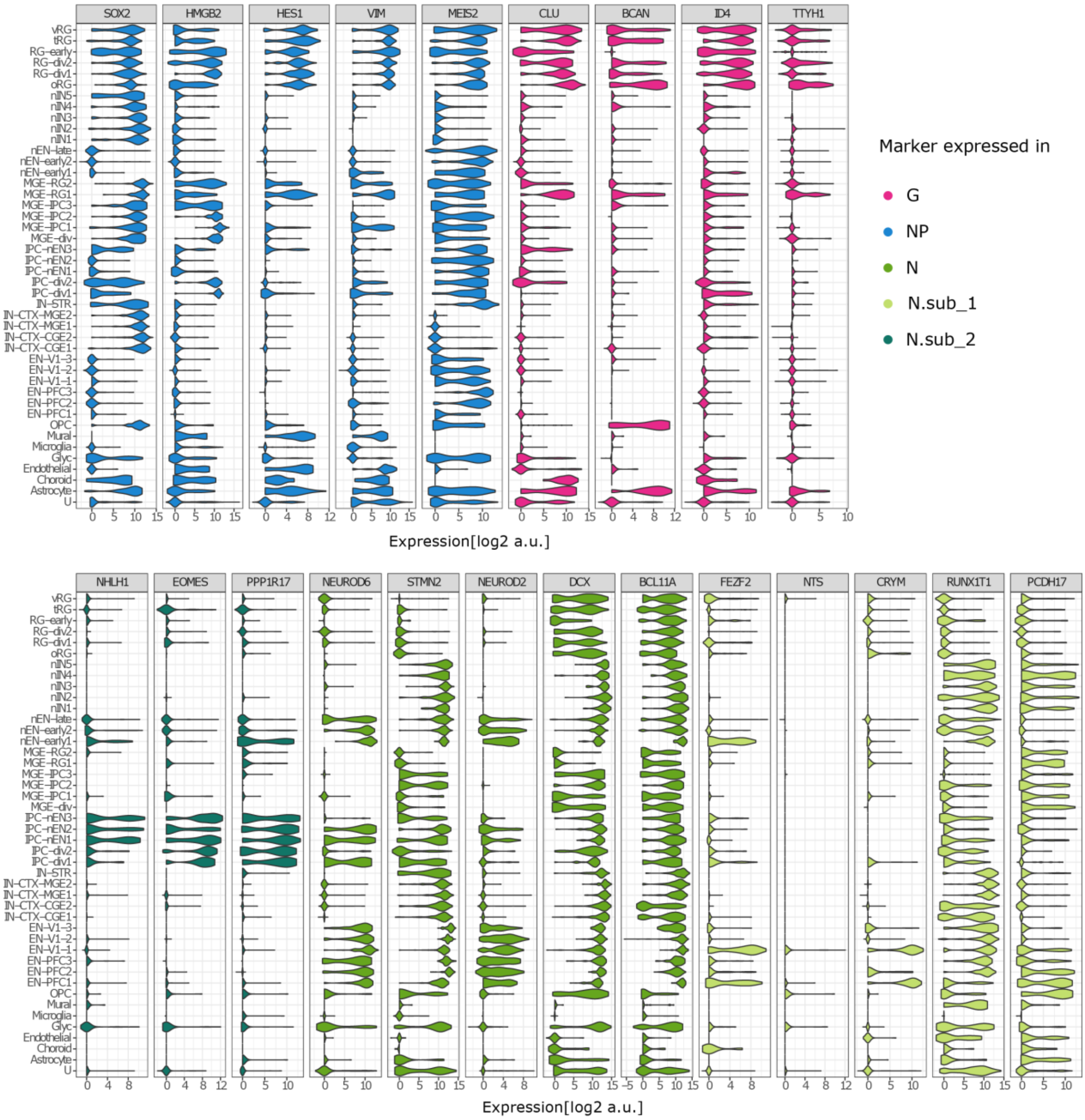


**Figure S7:** Comparison of neuronal population markers to scRNA-seq data from the developing human cortex. The data plotted are from a recent publication by Nowakowski *et al.*[1] . Shown are normalized expression values on a log2-scale, separated by cell type annotation as provided by the authors. For each gene, colors indicate which population expresses it highest in the in vitro corticogenesis model presented in this study. Abbreviations: RG = radial glia, vRG = ventricular RG, tRG = RG = radial glia, oRG = outer RG, IN = interneuron, nIN = newborn IN, EN = excitatory neuron, nEN = newborn excitatory neuron, MGE = medial ganglionic eminence, CGE = caudal ganglionic eminence, STR = striatum, CTX = cortex, V1 = primary visual cortex, PFC = prefrontal cortex, OPC = oligodendrocyte precursor cell, U = unknown.

**Table S1:** Composition of full and subsampled cell line datasets.

| | Full dataset | | Subset 1 | | Subset 2 | |
|---|---|---|---|---|---|---|
| | ***Number*** | ***%*** | ***Number*** | ***%*** | ***Number*** | ***%*** |
| A549 | 1320 | 11.3 | 400 | 8.0 | 80 | 2.0 |
| H1437 | 1116 | 9.6 | 270 | 5.4 | 3 | 0.08 |
| HCT116 | 1743 | 14.9 | 1400 | 28.0 | 1599 | 40.1 |
| HEK293 | 2002 | 17.1 | 1600 | 32.0 | 2000 | 50.2 |
| IMR90 | 1039 | 8.9 | 500 | 10.0 | 100 | 2.5 |
| Jurkat | 962 | 8.2 | 100 | 2.0 | 6 | 0.15 |
| K562 | 1604 | 13.7 | 379 | 7.6 | 70 | 1.8 |
| Ramos | 1892 | 16.2 | 350 | 7.0 | 125 | 3.1 |
| ***TOTAL*** | **11678** | **100** | **4999** | **100** | **3983** | **100** |

**Table S2:** Overview of clustering algorithms benchmarked in this study.

| Method | Unsupervised # clusters? | Input | Underlying model | Expected cluster shape and size[1] | Run time[2] |
|---|---|---|---|---|---|
| **SC3** [2] | Yes | Normalized data as an SCESet, distances and transformations are calculated internally | K-means clustering on various distances & transformations, hierarchical clustering of consensus matrix | Spherical, equal sizes | 35 min (using hybrid SVM approach) |
| **Hclust + dynamic tree cut** [3,4] | No | Pearson or euclidean distance in PCA space | Agglomerative clustering | None | 1 min |
| **pcaReduce** [5] | No | Normalized data, PCA is performed internally | K-means + hierarchical clustering | Spherical, equal sizes | 3 min |
| **Seurat** [6] | Yes | Normalized, log2 transformed counts as a Seurat object | Graph based | None | 9 min |
| **MCL** [7,8] | Yes | Pearson distance in PCA space | Graph based | None | Build graph: >1h Run MCL: 7 min |
| **mclust** [9] | Yes (via cross-validation or BIC) | Principal component scores | Gaussian mixture model | Ellipsoid, size can vary | 6 min |
| **DBScan** [10,11] | Yes | Euclidean distance in PCA space | Clusters are defined as regions of high density separated by regions of low density | None, but clusters have to be compact and clearly disconnected | 2 min |

[1] By size, we are referring to the actual distribution of the points in space, NOT the number of points in the cluster. For a Gaussian ellipsoid, size is parameterized by the covariance matrix.
[2] Run time was estimated using the system.time() function in R. The time shown here refers to the full dataset (12000 cells). Analysis was run on 64-bit Intel(R) Xeon(R) CPU E7-4850 v2 @ 2.30GHz with 1TB of RAM in R 3.4.1 under Red Hat Enterprise Linux Server release 6.9 (Santiago). SC3 was run on 8 cores, all other methods on a single core.

**Table S3:** Media composition for the in-vitro differentiation of cortical excitatory neurons from human pluripotent stem cells in suspension**.**

| Media name | Composition |
|---|---|
| **Phase I** | Advanced DMEM/F12 (Gibco, 12634010), GlutaMax (Gibco, 35050061) 1% v/v, Pen/Strep (Gibco, 10378016) 1% v/v, N-acetyl-cysteine (Sigma, A9165) 500µM, Heparin (Sigma, 375795) 2 µg/mL, SB431542 (Tocris, 1614/1) 10 µM, LDN193189 (Tocris, 6053/10) 100nM, XAV939 (Tocris,3748/10) 2µM, N2 Supplement (Gibco, A1370701) 0.5% (v/v). |
| **Phase II** | Advanced DMEM/F12, GlutaMax 1% v/v, Pen/Strep 1% v/v, N-acetyl-cysteine 500µM, Heparin 2 µg/mL, N2 Supplement 0.5% v/v, B27 Supplement (Gibco, 17504044) 1% v/v, FGF2 10ng/mL (first 4 days)/2.5ng/mL (rest of PhII), LDN193189 100nM, CHIR99021 (Tocris, 4953/10) 20nM, Retinoic Acid (Sigma, R2625) 5nM. |
| **Phase III** | Advanced DMEM/F12, GlutaMax 1% v/v, Pen/Strep 1% v/v, Heparin 2 µg/mL, N2 Supplement 0.5% v/v, B27 Supplement 0.4 % v/v, Forskolin (Tocris, 1099/10) 10 µM, CaCl2 0.6 mM. |
| **Phase IV** | Advanced DMEM/F12, GlutaMax 1% v/v, Pen/Strep 1% v/v, Heparin 2 µg/mL, N2 Supplement 0.5% v/v, B27 Supplement 1.0 % v/v, Forskolin 10 µM, CaCl2 0.6mM, BDNF (Peprotech, AF-450-02) 5.0 ng/mL, GDNF (Peprotech, AF-450-10) 5.0 ng/mL. |

**Table S5:** Sequencing statistics and QC cutoffs per batch.

| | **Batch 1: IMR90, HCT116** | **Batch 2: HEK293, H1437** | **Batch 3: A549, Ramos** | **Batch 4: K562, Jurkat, Ramos** | **Neuronal differentiation** |
|---|---|---|---|---|---|
| Reads / cell [mean] | 37'756 | 33'589 | 38'602 | 36'236 | 59'660 |
| UMI / cell [min, 1st quartile, **median,** 3rd quartile, max] | 4569,14224,**17869,** 23196, 64864 | 4774, 9844, **12960**, 19469, 88332 | 4377, 9774, **13543**, 22310, 56086 | 1009, 8896, **16600**, 23824, 78355 | 2827, 5044, **6989**, 9474, 76642 |
| Genes / cell [min, 1st quartile, **median,** 3rd quartile, max] | 900, 3166, **3646,** 4200, 7344 | 1010, 2838, **3278**, 3870, 9211 | 1303, 2556, **3142**, 4004, 6608 | 168 ,2550, **3652**, 4357, 6852 | 1371, 2084, **2606**, 3135, 7131 |
| Filter thresholds | Min_UMI: 2^13<br>Min_genes: 2^11<br>Max_pct_mt: 10 | Min_UMI: 2^12<br>Min_genes: 2^10.5<br>Max_pct_mt: 10 | Min_UMI: 2^12<br>Min_genes: 2^10.5<br>Max_pct_mt: 10 | Min_UMI: 2^12<br>Min_genes: 2^10.5<br>Max_pct_mt: 10 | Min_UMI = 2^11.5<br>Min_genes = 2^10.5<br>Max_pct_mt = 10 |
| % cells removed by RNA content filter | 6 | < 1 | < 1 | 3 | 17 |
| % cells removed by mt genes filter | 4 | < 1 | < 1 | < 1 | 15 |
| Total % cells removed | 9 | < 1 | < 1 | 3 | < 1 |
| Number of genes x cells passing QC | 9452 x 2823 | 9858 x 3274 | 9290 x 3118 | 8727 x 2701 | 10351 x 4857 |
| Cell type assignment | IMR90: 1039<br>HCT116: 1743<br>Doublet: 38<br>Unassigned: 3 | H1437: 1116<br>HEK293: 2002<br>Doublet: 0<br>Unassigned: 156 | A549: 1320<br>Ramos: 1769<br>Doublet: 29<br>Unassigned: 0 | K562: 1606<br>Jurkat: 962<br>Ramos: 123<br>Doublet: 0<br>Unassigned: 0 | n.a. |

**References**

1. Nowakowski TJ, Bhaduri A, Pollen AA, Alvarado B, Mostajo-Radji MA, Di Lullo E, et al. Spatiotemporal gene expression trajectories reveal developmental hierarchies of the human cortex. Science (80- ) [Internet]. 2017;358:1318–23. Available from: http://www.ncbi.nlm.nih.gov/pubmed/29217575

2. Kiselev VY, Kirschner K, Schaub MT, Andrews T, Yiu A, Chandra T, et al. SC3: Consensus clustering of single-cell RNA-seq data. Nat Methods. 2017;14:483–6.

3. Langfelder P, Zhang B, Horvath S. Dynamic Tree Cut : in-depth description , tests and applications. Bioinforamtics. 2007;1–12.

4. Langfelder P, Zhang B, Horvath S. Defining clusters from a hierarchical cluster tree: The Dynamic Tree Cut package for R. Bioinformatics. 2008;24:719–20.

5. žurauskiene J, Yau C. pcaReduce: Hierarchical clustering of single cell transcriptional profiles. BMC Bioinformatics. 2016;17.

6. Macosko EZ, Basu A, Satija R, Nemesh J, Shekhar K, Goldman M, et al. Highly parallel genome-wide expression profiling of individual cells using nanoliter droplets. Cell. 2015;161:1202–14.

7. Enright AJ, Van Dongen S, Ouzounis CA. An efficient algorithm for large-scale detection of protein families. Nucleic Acids Res [Internet]. 2002;30:1575–84. Available from: http://www.ncbi.nlm.nih.gov/pubmed/11917018

8. Stijn van Dongen. Graph Clustering by Flow Simulation. University of Utrecht; 2000.

9. Fraley C, Raftery AE. Model-based Clustering, Discriminant Analysis and Density Estimation. J Am Stat Assoc. 2002;97:611–31.

10. Ester M, Kriegel HP, Sander J, Xu X. A Density-Based Algorithm for Discovering Clusters in Large Spatial Databases with Noise. Proc 2nd Int Conf Knowl Discov Data Min [Internet]. 1996;226–31. Available from: https://www.aaai.org/Papers/KDD/1996/KDD96-037.pdf

11. Campello RJGB, Moulavi D, Sander J. Density-Based Clustering Based on Hierarchical Density Estimates. Adv Knowl Discov Data Min [Internet]. 2013;160–72. Available from: http://link.springer.com/10.1007/978-3-642-37456-2_14
