# Supplementary material for: CellSIUS provides sensitive and specific detection of rare cell populations from complex single-cell RNA-seq data
Source: Genome Biol. 2019 Jul 17;20:142. doi: 10.1186/s13059-019-1739-7 (PMC6637521; doi:10.1186/s13059-019-1739-7)
Supplement: Supplementary file 3 — Review history (DOCX 58 kb) [file 13059_2019_1739_MOESM3_ESM.docx]

Review History

**First round of review**

**Reviewer 1**

**Were you able to assess all statistics in the manuscript, including the appropriateness of statistical tests used?**

**Yes**: This is a methodological paper and statistics assessment is part of the review.

**Were you able to directly test the methods?**

No

**Comments to author:**

The manuscript proposes a cell type identification approach with a particular focus on rare cell type. The proposed method is well written and easy to follow, they provide an alternative to current rare cell type identification algorithm raceID and GiniClust. The key difference is in the second assignment step where CellSIUS restrict candidate marker genes to be cluster specific. The evaluation is based on both synthetic and real data. The evaluation component in this manuscript could be improved. Some major comments follow:

1) Should raceID and Giniclust be included in Table 1 for benchmarking? Is there further motivation other than making candidate marker genes more cluster specific and what is the direct impact of this particular component in rare cell identification.
2) Conceptually, rare cell type could be better identified via supervise learning or matching algorithm. Could the authors comment on how CellSIUS compares with such classes of methods such as Moana, scAlign and scMaps and many others. 
3) There are various parameters decision in CellSIUS (Page 36 - 37) such as 2-fold cutup for candidate maker (in step 1); the initial value m, the threshold values of 0.35 and 5 for correlation (in step 3); the value min_n_gene (in step 5) and others. A brief sensitivity analysis of the impact of these various parameters on the cell type identification outcome will be useful. 
4) I am not fully convinced by the evaluation data that CellSISU provide an substantially improve from raceID. Not sure if the Figure 4A is schematic or real data. It shows very strong signal between the 3 cell types. Using a more complex example may provide a better illustration of how robust is the CellSIUS to different types of single cell data. Complexity could be subsampling from more cell types or subsampling from cell type with more correlated (or overlapping) markers. 
5) I found other comparison metric may be more transparent. For example, adjust rand index or given this is rare cell type, the number of correctly identify cell (given this number is so small) be a more informative in comparison to a percentage metric (such as precision and recall). 
6) For subtlety (swapped genes) = 1 on the graph, no algorithm should be able to detect the rare cell. This is not clear from Figure 4B or 4C. Is this the case?

**Reviewer 2**

**Were you able to assess all statistics in the manuscript, including the appropriateness of statistical tests used?**

**Yes**: There is very little in the way of statistical reasoning in this manuscript - where required I have highlighted the need for greater justification or detail.

**Were you able to directly test the methods?**

No

**Comments to author:**

Generally speaking the manuscript is somewhat incoherent and lacks sufficient detail to make an informed judgement of the method. The requirement for another "rare cell" identification method uses a strawman argument and is not realistic. The generation of a high-quality and well-defined dataset for benchmarking is commendable, however, its use as a motivation for developing CellSIUS is artificial.

The clustering benchmarking is not reflective of commonly used methods for single-cell experiments. There should be a greater coverage of graph-based methods, including different community detection algorithms and how these perform for the identification of low-frequency cell populations. Additionally, such a benchmarking should detail the effects of varying parameters such as the size of the cell neighbourhood for graph construction, as well as the community size for cluster detection.

The inclusion and description of the hPSC-derived neural differentiation data seems more fitting for a neuroscience journal - it is not clear what the added value for the manuscript or the method is beyond a more realistic data set. There exist a multitude of publicly available datasets that are more than sufficient for such a comparison.

Specific comments
Line 22 - "…over one hundred thousand cells…" - the first million-cell single-cell data sets have now started to emerge thus this sentence is out of data.

Line 36 - "…feature reduction…"; commonly referred to as feature selection.

Lines 42-43 - "…rare cell populations (<0.16%)" - this is a very strange and arbitrary %. There is no consensus on what constitutes a "rare" cell population.

Lines 78-88 - This paragraph is better suited to the methods section; it is not a result.

Line 108 - provide an explicit justification for using NBDrop for feature selection - one assumes this is because it selects genes that explain more of the cell-line based variance.

Line 149 - justify the use of graph-based clustering to identify correlated gene sets.

Lines 163-170 - Why generate simulations when you have generated a good benchmarking data set? Furthermore, why not use a more accurate method for simulating data, such as BASiCs (Vallejos et al. PLoS Comp Bio - 2015) or Splatter (Zappia et al. Genome Biology -2017)?

Alternatively, one could use the cell line data, and spike in a specific cell line at different proportions as a more accurate comparison. This is similar to the comparison presented in Figure 4 but without sequentially merging gene expression profiles.

Line 195 - what does it mean that cells are "perturbed"? This implies and experimental intervention which is clearly not the case here.

Lines 193-207 - This comparison is somewhat incoherent and would benefit greatly from a schematic in a supplementary figure.

Lines 210-211 - Why restrict to cells in G1? This creates a very artificial scenario. As you can classify these cells you can validate whether any rare cell populations recovered represent different cell cycle phases.

Line 231 - this is the first mention of MCL as the graph-based clustering prior to CellSIUS - this needs to be mentioned much earlier. Furthermore, there should be a comparison or recommendation for the clustering prior to CellSIUS.

Lines 239-241 - I wouldn't describe 3 genes as a "signature", it could just as well be a technical artifact, such as a multiplet from droplet-based scRNA-seq methods, or a contamination cell type.

Lines 243 - 262- The description of the differentiation protocol belongs in the methods. Furthermore, there is no description of what the single-cell RNA-sequencing data are, i.e. SMART-Seq, 10X Chromium, Drop-seq, etc?

If these data are droplet-based then the authors need to unequivocally demonstrate that the "rare cell" populations they identify are not doublets.

Lines 276-277 - this is an arbitrary threshold value with no justification. What statistical test was this p-value calculated from?

Lines 284 - Reference to Figure 6F is incorrect, this shows a contingency that is not described - I assume the authors mean Figure 6E? Furthermore, Figure 6F is not described in the manuscript anywhere.

Lines 279-322 - more unnecessary description of the differentiation protocol, this should be moved to the methods as it has little-to-no relevance for the clustering method presented.

Line 329 - There is no Figure 7.

Lines 355-358 - The description of the different methods comparison is built on the assumption that CellSIUS represents the ground truth - this is fundamentally incorrect and should be changed to reflect this.

Methods - there are two different versions of the human genome and cellranger pipeline used - these should be the same across all analyses.

Lines 640; methods - what is the effect of varying the mean log fold change on simulations and clustering performance?

Lines 654-655; methods - define "subtlety" parameter.

Lines 682; methods - these parameter choices are quite arbitrary and lack justification.

Lines 710-717; methods - the CellSIUS method essentially relies on the ability to detect multi-model gene expression patterns within a cluster. A k-means partitioning on the log2(normalised counts +1) is almost certainly guaranteed to find a population of 0's and non-zero expression. This is an artificial multi-modality caused by the log2(counts +1) transformation - see https://www.biorxiv.org/content/10.1101/574574v1 and https://www.biorxiv.org/content/10.1101/576827v2 for details.

Lines 730-732; methods - this sentence is incomprehensible, a schematic or more formal definition may clarify what is meant here.

**Authors Response**

**Point-by-point responses to the reviewers’ comments:**

Reviewer reports:

Reviewer #1: The manuscript proposes a cell type identification approach with a particular focus on rare cell type. The proposed method is well written and easy to follow, they provide an alternative to current rare cell type identification algorithm raceID and GiniClust. The key difference is in the second assignment step where CellSIUS restrict candidate marker genes to be cluster specific. The evaluation is based on both synthetic and real data. The evaluation component in this manuscript could be improved. Some major comments follow:

*We thank the reviewer for this review and constructive criticism. We believe we have addressed all the major concerns of the reviewer below.*

1) Should raceID and Giniclust be included in Table 1 for benchmarking?

*Whilst interesting and complementary to our work, clustering benchmarking is not within the scope and aim of our paper. In this context, a recent publication in Nature Reviews Genetics describes challenges in unsupervised clustering of single-cell RNA-seq data and is greatly complementary to the our manuscript (Kiselev et al., 2019, published concurrently with our submission to Genome Biology). In particular, one of the overall conclusions is consistent with the starting point of our paper highlighting the methodology gap for robust and specific rare cell type identification:*

*“Many methods work best when clusters are approximately equal in size, tools such as GiniClust and RaceID have been specifically tailored to identify rare cell types. Unfortunately, a better ability to distinguish rare cell types comes at the cost of poorer performance when clustering more frequent cell types”.*

*Consequently, we have adapted and minimized the sections on the feature selection methods for common cell types as well as he clustering algorithm evaluation to concentrate on strengthening the central message, i.e. CellSIUS and rare cell type identification (see additional work to address the reviewer points 3 and 4). In addition to the changes in the text, we removed Tables S1 and moved Table 1 to the supplementary material.*

Is there further motivation other than making candidate marker genes more cluster specific and what is the direct impact of this particular component in rare cell identification?

*Making candidate marker genes cluster specific comes with the promise to overcome some technical biases that affect all clusters equally, e.g. different number of detected genes between cells, differences in the total number of counts per cell or normalization artifacts and has the potential to help disentangle technical and biological variability and increase the precision of rare cell type identification.*

2) Conceptually, rare cell type could be better identified via supervise learning or matching algorithm. Could the authors comment on how CellSIUS compares with such classes of methods such as Moana, scAlign and scMaps and many others.

*Supervised learning approaches entail presenting labelled learning instances to an algorithm in order for it to learn to predict the label of new test instances. In the case of single cell RNA sequencing data this would equate to training a method with transcriptomes of known cell types. Crucially, any such method requires labelled training data. Conceptually simpler approaches based on correlations of single cell RNA sequencing data to labelled training data are also employed, as demonstrated by a recent publication focusing on profibrotic lung macrophages and implemented in the R package singleR. [Aran, D. et al. Reference-based analysis of lung single-cell sequencing reveals a transitional profibrotic macrophage. Nature Immunology 2017 18:5 20, 163–172 (2019).] Of the three algorithms mentioned by the reviewer, scmap is closely related to singleR, but scmap takes a consensus vote approach employing three different distance measures between test and reference cells. Moana is a hierarchical machine learning method that uses chained support vector machines (SVMs) to predict the identity of cells at different levels of granularity. Finally, scAlign is an unsupersived method aimed at data integration through the learning of a common (low-dimensional) embedding of datasets coupled with the incorporation of cell-type specific information; the aim is to allow same (or similar) cell types to be identified in different datasets. Any rare cell type(s) identified in this way is (are) the result of the inability to match a cell type across two (or more) datasets.*

*In contrast to all of these methods, CellSIUS aims to identify sets of cells within clusters of a single cell RNA sequencing experiment in a way that these cells share a common set of expressed genes that no other group of cells does. Whereas scmap and scAlign try to bridge across datasets, CellSIUS is aimed to work with one dataset; indeed, the result of scAlign might very well be used as the input to a subsequent CellSIUS analysis. While CellSIUS will identify the existence of rare cell types, it will not label them; therefore, Moana (and methods such as singleR) might be appropriate tools to further characterize such rare cell types.*

*Overall, the problems addressed by CellSIUS as opposed to scmap, scAlign, and moana are quite different, and the solutions they provide are complementary.*

3) There are various parameters decision in CellSIUS (Page 36 - 37) such as 2-fold cutup for candidate maker (in step 1); the initial value m, the threshold values of 0.35 and 5 for correlation (in step 3); the value min_n_gene (in step 5) and others. A brief sensitivity analysis of the impact of these various parameters on the cell type identification outcome will be useful.

*The reviewer asked for a sensitivity analysis of the impact of CellSIUS parameters on the rare cell type identification outcome, which makes a lot of sense. We carried out such an analysis and included the results in the text (lines ...) as well as in supplementary Figure S3. In addition, we provide the reviewer with a HTML report of this work.*

*Briefly: both sensitivity and specificity are stable across a wide range of combinations of the key parameters, except for stringent corr_cutoff of 0.95, confirming the robustness of the algorithm.*

*Recommendation for the users: even if the values of the parameters depend on the data, we suggest using the default parameters, at least for the first exploration. In addition, fc_between should not be set higher than fc_within. In general, corr_cutoff=NULL (default value resulting in a data-driven selection of the cutoff) works better than forcing a fixed value. We had provided recommendations in the R package documentation to guide the users on how to use some of these parameters in the original submission.*

4) I am not fully convinced by the evaluation data that CellSIUS provide a substantially improve from raceID.

*Comparison of the performance of CellSIUS to existing approaches for identification of rare cell types and outlier genes (RaceID3 and GiniClust2) in both synthetic and biological data clearly highlighted the superiority of our approach for both specificity and selectivity. E.g., in the cortex neuron data, a complex and heterogeneous dataset, only CellSIUS was able to identify the rare choroid plexus (CP) lineage, for which we experimentally validated the expression of the identified cell subtype markers at the protein level. In addition, CellSIUS is specifically designed to identify only cluster substructures: this allows the user to choose the most appropriated clustering algorithm according to the data complexity for a main cell-type identification. In contrast the RaceID and GiniClust rare cell-type performances could be affected and biased by the integrated clustering approach for frequent cell-type selection, that could be suitable for some dataset but not for others.*

Not sure if the Figure 4A is schematic or real data. It shows very strong signal between the 3 cell types. Using a more complex example may provide a better illustration of how robust is the CellSIUS to different types of single cell data. Complexity could be subsampling from more cell types or subsampling from cell type with more correlated (or overlapping) markers.

*The reviewer raised the question whether the data in figure 4A were real or schematic. Figure 4A is schematic data, and the differences between the cell types are exaggerated to ease visualization. We clarified this in the figure caption.*

*Furthermore, the reviewer was concerned that the transcriptional differences between the cell types used for benchmarking were not complex enough to illustrate the validity of CellSIUS. To address this point, we provide the reviewer with a HTML report for visualization of the transcriptional signatures of the cell types used for the benchmarking analysis displayed in Figure 4 B and C. Unlike the schematic depiction in figure 4A, the transcriptional profiles of these cell types show a high degree of correlation: at a subtlety value of 0.995 only 0.5% of the genes are different between the rare cell type and its closest neighbor. We therefore think the data we used is complex enough to provide an illustration of how robust CellSIUS is and this was clarified in the text (lines ...). The heatmaps from the HTML report could be added as a supplementary figure if the reviewer/editor judge this is necessary in addition to the text clarification.*

5) I found other comparison metric may be more transparent. For example, adjust rand index or given this is rare cell type, the number of correctly identify cell (given this number is so small) be a more informative in comparison to a percentage metric (such as precision and recall).

   *The adjusted rand index (ARI) works very well for cases where clusters are roughly equally sized. However, if one of the clusters is very small as is the case here, the ARI will always be close to 1 as long as the two big clusters are identified correctly, making it less well suited for this comparison.*

*Recall, or the fraction of correctly identified rare cells, is directly proportional to the number of correctly identified cells: Recall is defined as the number of correctly identified rare cells divided by the total number of rare cells. We have clarified this relationship in the figure legend and in the main text. We decided to use recall instead of the raw umber of correctly identified cells because it provides a common scale across any number of rare cells. A recall of 1.0 is equivalent to saying n out of n rare cells were identified correctly.*

*Precision, on the other hand, measures how many of the cells an algorithm calls rare cells are actually rare cells. It is again directly related to the total number of identified rare cells, since precision = (number of falsely identified rare cells + number of correctly identified rare cells) / total number of rare cells.*

*Based on the reviewer’s comment we added the raw number cell numbers to the text throughout.*

6) For subtlety (swapped genes) = 1 on the graph, no algorithm should be able to detect the rare cell. This is not clear from Figure 4B or 4C. Is this the case?

   *The reviewer pointed out that for a subtlety of 1.0, none of the algorithms should identify any rare cells. We thank the reviewer for this comment, and we adjusted the legend in Figures 4 B and C to clarify that the maximum value of subtlety we used was 0.995, not 1.0. In the attached HTML report, we show that indeed for a value of 1.0 none of the algorithms would pick up the “rare cell type”. Notably, GiniClust2 and RaceID3 split the main clusters whereas CellSIUS does not.*

Reviewer #2:

*We thank the reviewer for this extensive review and constructive criticism. We believe we have addressed all the major concerns of the reviewer below.*

Generally speaking the manuscript is somewhat incoherent and lacks sufficient detail to make an informed judgement of the method. The requirement for another "rare cell" identification method uses a strawman argument and is not realistic. The generation of a high-quality and well-defined dataset for benchmarking is commendable, however, its use as a motivation for developing CellSIUS is artificial.

The clustering benchmarking is not reflective of commonly used methods for single-cell experiments. There should be a greater coverage of graph-based methods, including different community detection algorithms and how these perform for the identification of low-frequency cell populations. Additionally, such a benchmarking should detail the effects of varying parameters such as the size of the cell neighbourhood for graph construction, as well as the community size for cluster detection.

*The first paragraph of reviewer 2 was somewhat surprising to us, yet duly considered; our response is based on an objective analysis and not intended to be construed as an exercise in sophism. We share the concern of the reviewer that generating data for the sake of more data is not in and of itself always a worthwhile exercise. Regarding the strawman argument, we however need to return this to the reviewer as she/he clearly fell victim to it: as stated in the manuscript, the aim was “[…] to investigate the performance of scRNA-seq feature selection and clustering approaches […]”, the results of which “[…] highlighted a methodology gap for sensitive and specific identification of rare cell types”. In other words, the generation of the dataset was not the motivation for the development of CellSIUS. In contrast, the reviewer claims “The generation of a […] dataset for benchmarking is commendable, however, its use as a motivation for developing CellSIUS is artificial“. Therefore, the actual strawman argument is provided by the reviewer, not by us. At the same time, it is somewhat contradictory to commend the generation of a (otherwise absent from the literature/public domain) benchmarking dataset and at the same time label its use artificial; what is not commendable (and can be truly artificial) is the benchmarking of algorithms on artificial data and/or not well-defined experimental datasets.*

*Whilst interesting and complementary to our work, extensive clustering benchmarking is not within the scope and aim of our manuscript, as pointed out in the previous paragraph. In this context, a recent Nature Reviews Genetics publication describes challenges in unsupervised clustering of single-cell RNA-seq data and is greatly complementary to our manuscript (Kiselev et al., 2019, published concurrently with our submission to Genome Biology). In particular, one of the overall conclusions is consistent with the starting point of our paper highlighting the methodology gap for robust and specific rare cell type identification:*

*“Many methods work best when clusters are approximately equal in size, tools such as GiniClust and RaceID have been specifically tailored to identify rare cell types. Unfortunately, a better ability to distinguish rare cell types comes at the cost of poorer performance when clustering more frequent cell types”.*

*Consequently, we have adapted and minimized the sections on the feature selection methods for common cell types as well as the clustering algorithm evaluation to concentrate on strengthening the central message, i.e. CellSIUS and rare cell type identification (see additional work to investigate CellSIUS’ sensitivity and specificity across a wide range of combinations of the key parameters). In addition to the changes in the text, we removed Tables S1 and moved Table 1 to the supplementary material.*

The inclusion and description of the hPSC-derived neural differentiation data seems more fitting for a neuroscience journal - it is not clear what the added value for the manuscript or the method is beyond a more realistic data set. There exist a multitude of publicly available datasets that are more than sufficient for such a comparison.

*We thank the reviewer for his/her comment and acknowledge there exist a multitude of publicly available scRNA-seq data sets. For the choice of our benchmarking datasets, we strictly adhered to Genome Biology Method section recommendations, including synthetic data, generating a 12,000 cells dataset where the ground truth is known and a highly complex real dataset of 5,000 cell. The latter represents a well-characterized cellular system that we use for internal research that came with the possibility to access the original samples for follow-up experimental validation. For all of those datasets, we clearly demonstrated the utility and superiority of CellSIUS over other existing methods, with experimental validation on the protein level of our algorithm prediction in our highly complex real dataset.*

*The aim behind the choice of datasets featured in our manuscript was to hold ourselves to similarly high standards as the methods that we compare to: in line with this, the publication of RaceID3 (Herman, J. S., Sagar & Grün, D. FateID infers cell fate bias in multipotent progenitors from single-cell RNA-seq data. Nature Publishing Group 15, 379–386 (2018).) also included newly generated datasets for the development of the algorithm as well as assays for validation of the computational results. Evidenced by this example, journal guidelines as well as reviewer’s opinions do differ; further to that, we are convinced that, if anything, the additional validation enabled by the neural differentiation data adds to the value of our manuscript, as opposed to diluting the content or lessen its value for the research community.*

Specific comments

Line 22 - "…over one hundred thousand cells…" - the first million-cell single-cell data sets have now started to emerge thus this sentence is out of date.

*Line 22: wording was change to “.. more than one million cells...”*

Line 36 - "…feature reduction…"; commonly referred to as feature selection.

*Line 36 and 361: wording was changed to “...feature selection..”.*

Lines 42-43 - "…rare cell populations (<0.16%)" - this is a very strange and arbitrary %. There is no consensus on what constitutes a "rare" cell population.

*Lines 42-43, we removed (<0.16%) from this section as this does seem out of place referring to a specific results that appears in the result section at a later point.*

Lines 78-88 - This paragraph is better suited to the methods section; it is not a result.

*The workflow description is now minimal and refers to the methods section where this paragraph was moved (lines 614-625).*

Line 108 - provide an explicit justification for using NBDrop for feature selection - one assumes this is because it selects genes that explain more of the cell-line based variance.

*Line 99-101: Added explicit justification: ..” because NBDrop selected genes explain more cell-line based variance, we compared some of the most recent or widely used clustering methods after feature selection using NBDrop...”*

Line 149 - justify the use of graph-based clustering to identify correlated gene sets.

*We applied the Markov Cluster Algorithm (MCL), a graph-based clustering algorithm. One of the main motivations for choosing MCL was the absence of a prespecified number of clusters as required for other approaches such as k-means. Practically, in our setting MCL works on the gene correlation network derived from single cell RNAseq data and detects communities in this network. These (gene) communities are guaranteed to contain genes that are co-expressed, by design. In contrast, in a k-means clustering with a pre-specified k we cannot be sure that all genes within all clusters are co-expressed to the same degree: genes are assigned the closest centroid, but this does is only a relative measure. Thus, by using communities of a gene correlation network, with a pre-specified correlation threshold, we can be sure that those communities (if such exist) satisfy the criteria of containing correlated genes. Overall, no parameter for number of clusters, computational speed, and ease of use made MCL a straightforward choice for the problem at hand; moreover, the resulting transcriptional co-expression networks (might) lend themselves to biological interpretation. Based on the reviewer’s comment, we have clarified the use of MCL in the text (lines ....).*

Lines 163-170 - Why generate simulations when you have generated a good benchmarking data set?

*We strictly adhered to Genome Biology Method section recommendations regarding the dataset used for benchmarking of our approach. See response to point #2.*

Furthermore, why not use a more accurate method for simulating data, such as BASiCs (Vallejos et al. PLoS Comp Bio - 2015) or Splatter (Zappia et al. Genome Biology -2017)?

*Lines 163-170: Based on the reviewer’s comment we re-generated the simulated data using Splatter (Zappia et al. Genome Biology -2017) and we provide the reviewer with a HTML report showing that our approach generates simulated data with mean-variance and mean-dropout relationships, library sizes and percentage of zero counts per cells and per gene that is more similar to the underlying real data than the simulated data generated by Splatter. We therefore did not adapt this part of the manuscript.*

Alternatively, one could use the cell line data, and spike in a specific cell line at different proportions as a more accurate comparison. This is similar to the comparison presented in Figure 4 but without sequentially merging gene expression profiles.

*With a benchmark dataset of known composition and synthetic data at hand, we do not feel this is necessary. As mentioned by the reviewer, we carried out a similar approach in Figure 4. In addition, in Figure 3, we benchmark both the marker genes and the rare cell types.*

Line 195 - what does it mean that cells are "perturbed"? This implies and experimental intervention which is clearly not the case here.

*We added the following clarification: “ ... Briefly, 20 cells (~2% of total cells) were added in one cluster upregulating between 2 and 100 genes by a log2 fold change of 2.5 on average...” (line xxx)*

Lines 193-207 - This comparison is somewhat incoherent and would benefit greatly from a schematic in a supplementary figure.

*We replace this section by a more comprehensive sensitivity analysis of the impact of CellSIUS parameters on the rare cell type identification outcome as discussed above and clarified the text (lines ...).*

Lines 210-211 - Why restrict to cells in G1? This creates a very artificial scenario. As you can classify these cells you can validate whether any rare cell populations recovered represent different cell cycle phases.

*By selecting only G1-phase cells, we ensure the homogeneity and validity of our benchmark dataset. One of the reviewer congratulated us on our level of thoughtfulness and meticulous attention to detail for this point.*

Line 231 - this is the first mention of MCL as the graph-based clustering prior to CellSIUS - this needs to be mentioned much earlier. Furthermore, there should be a comparison or recommendation for the clustering prior to CellSIUS.

*Single cell RNA-seq dataset are highly heterogenous and the performance of single cell algorithms can vary quite dramatically across datasets. Our comparison of clustering approaches highlights that the choice of the clustering approach needs to be adapted to the nature of the considered scRNA-seq data. We now introduce MCL as our approach of choice for our clustering module much earlier (line 123) and refer to the recent Nature Reviews Genetics publication on challenges in unsupervised clustering of single-cell RNA-seq data for recommendation for the clustering prior to CellSIUS.*

Lines 239-241 - I wouldn't describe 3 genes as a "signature", it could just as well be a technical artifact, such as a multiplet from droplet-based scRNA-seq methods, or a contamination cell type.

*We agree with the reviewer that there are no generally accepted upper or lower bounds on how many members constitute a gene “signature”. Individual genes might be referred to as “markers” and abound in the biomedical literature. From two genes onwards, naming becomes idiosyncratic and inconsistent at best. As little as three genes are commonly referred to as “signature” in the literature (eg, Li, B. et al. Development and Validation of a Three-gene Prognostic Signature for Patients with Hepatocellular Carcinoma. Sci. Rep. 7, 5517 (2017).). In the absence of any objective criteria for what is and is not a “signature”, we would like to point out that for the two major signatures identified in the referenced text there are more than 3 genes.*

Lines 243 - 262- The description of the differentiation protocol belongs in the methods. Furthermore, there is no description of what the single-cell RNA-sequencing data are, i.e. SMART-Seq, 10X Chromium, Drop-seq, etc?

*Part of the differentiation protocol was moved to the method section. The single cell data was described in the method section in the original submission as generated using the 10X Genomics Chromium platform (line 550). This information was now also added to the main text as well for clarity (line 243).*

If these data are droplet-based then the authors need to unequivocally demonstrate that the "rare cell" populations they identify are not doublets.

*We would like to point out that there is a total absence in the field of any solution for post-hoc identification of doublet cells in single cell RNAseq data after already very stringently filtering.*

*Single cell RNA-seq data usually contain a small fraction of doublets, i.e. transcriptomes derived from two or more cells, which can form artifactual clusters. This should not be a concern here (e.g. Fig. S6A does not indicate the presence of doublet-driven clusters – each subcluster has its own unique markers). In addition, some of the rare cell populations we identify are either supported by literature (e.g. G.sub_1) or were validated experimentally. We have included this part in the paper’s discussion (lines....).*

Lines 276-277 - this is an arbitrary threshold value with no justification. What statistical test was this p-value calculated from?

*For the multiple test correction of the p value we used Benjamini-Hochberg, we have now specified in the text: “... using a cutoff of 1.3 for the standardized value, corresponding to a Benjamini-Hochberg corrected p adjusted < 0.05)...” (lines ...).*

Lines 284 - Reference to Figure 6F is incorrect, this shows a contingency that is not described - I assume the authors mean Figure 6E? Furthermore, Figure 6F is not described in the manuscript anywhere.

*Change to Figure 6E (line 280). Figure 6F is now described in the manuscript (lines ...).*

Lines 279-322 - more unnecessary description of the differentiation protocol, this should be moved to the methods as it has little-to-no relevance for the clustering method presented.

*The differentiation protocol as well as the functional and transcriptomic identity characterisation of our generated cortical neurons was moved to the methods section. In addition, Figure 5 was moved to supplementary material and merged with figure S4.*

Line 329 - There is no Figure 7.

*Due to reasons that we do not fully understand Figure 7 seemed not to have been uploaded during the submission to Genome Biology and is now included.*

Lines 355-358 - The description of the different methods comparison is built on the assumption that CellSIUS represents the ground truth - this is fundamentally incorrect and should be changed to reflect this.

*We draw conclusions based on the fact that the CP population we validated in lab has been correctly detected by CellSIUS and not by RaceID and GiniClust. In this respect, yes, we assumed CellSIUS is the ground truth since in this case it reflects the experimental evidences.*

Methods - there are two different versions of the human genome and cellranger pipeline used - these should be the same across all analyses.

*The commercial cell-lines and the neuronal directed differentiation have been single cell sequenced independently by different labs, one in Basel (CH) and one in Cambridge (US) using different human genomes. Since we don’t directly compared the two datasets, we strongly believe that does not affect the conclusion we have drown.*

Lines 640; methods - what is the effect of varying the mean log fold change on simulations and clustering performance?

*As mentioned before, detailed single cell clustering benchmark is beyond the aim of our study.*

Lines 654-655; methods - define "subtlety" parameter.

*We defined the “subtlety” parameter as subtlety the fraction of Jurkat genes replaced by Ramos genes in the text (lines ...).*

Lines 682; methods - these parameter choices are quite arbitrary and lack justification.

*A threshold cutoff is always arbitrary but there are some commonly accepted rules of thumb and Pearson coefficient of 0.2 can be consider proxy of low correlation.*

Lines 710-717; methods - the CellSIUS method essentially relies on the ability to detect multi-model gene expression patterns within a cluster. A k-means partitioning on the log2(normalised counts +1) is almost certainly guaranteed to find a population of 0's and non-zero expression. This is an artificial multi-modality caused by the log2(counts +1) transformation - see <https://urldefense.proofpoint.com/v2/url?u=https-3A__www.biorxiv.org_content_10.1101_574574v1&d=DwIGaQ&c=ZbgFmJjg4pdtrnL2HUJUDw&r=HW-PeCvV5D96_eAPwgTa4bKnS2K4oo3kO6geuiPyTN45OtwcGn2EJG0msmmTlrCl&m=tG9E6zuoKZqGzwdOosctX0LkLRFBaD4c61Ej4pE49YE&s=i1NuR6SpQeKv5OHOw309VbFCh-V2aC1DuXa5KYdbYSg&e>= and <https://urldefense.proofpoint.com/v2/url?u=https-3A__www.biorxiv.org_content_10.1101_576827v2&d=DwIGaQ&c=ZbgFmJjg4pdtrnL2HUJUDw&r=HW-PeCvV5D96_eAPwgTa4bKnS2K4oo3kO6geuiPyTN45OtwcGn2EJG0msmmTlrCl&m=tG9E6zuoKZqGzwdOosctX0LkLRFBaD4c61Ej4pE49YE&s=n_PaZT_UFdaD9GjQaryuDPmVbulzbDAdEzk9xGV99js&e>= for details.

*We assume that the reviewer means that expression counts are always multimodal, because they are discrete by nature. If you have log2 counts derived from discrete values, you will certainly find a hump at 0 and one at 1, just because gene counts can only take integer values.*

*We can argue that while this is certainly true for 1 and 0, by setting a default cutoff of 2 for the log2 fold change, we never consider this first modality. Besides, the 0 – 1 modality would be present in all clusters, so even if we did not have this first restriction, we would not consider such signal as meaningful because we require signatures to be cluster specific.*

Lines 730-732; methods - this sentence is incomprehensible, a schematic or more formal definition may clarify what is meant here.

We have simplified the sentence in the text: “The lower bound was set to 0.35. This value was chosen based on analysis of simulated datasets, where the gene-gene correlations of entirely homogeneous populations showed a maximum correlation value of 0.35” (line xxx)

**Second round of review**

**Reviewer 2**

The authors should be commended for greatly improving the clarity of their manuscript. I am confident that the major issues previously raised have been addressed.

I have 2 minor comments regarding a) the potential for doublets to increase the rare-cell detection false-positive rate and b) algroithm scalability due to the reliance on the MCL algorithm.

In the response to reviewers they state that no method exists to evaluate or identify droplets, however, 2 such methods currently exist (now published):

DoubletFinder - https://www.cell.com/cell-systems/fulltext/S2405-4712(19)30073-0?_returnURL=https%3A%2F%2Flinkinghub.elsevier.com%2Fretrieve%2Fpii%2FS2405471219300730%3Fshowall%3Dtrue

Scrublet - https://www.cell.com/cell-systems/fulltext/S2405-4712(18)30474-5?_returnURL=https%3A%2F%2Flinkinghub.elsevier.com%2Fretrieve%2Fpii%2FS2405471218304745%3Fshowall%3Dtrue

Whilst the authors have gone to stringent efforts to filter out doublets in their cell line based bench-marking analysis, no such details are provided for their single-cell neuronal data set. Whilst the authors validate the presence of the CP population experimentally, the assumption that the other rare cell groups that they identify are equally valid does not hold logically. The authors should demonstrate by one of the above methods that removing probable doublets does not appreciably alter their rare-cell population identification.

With respect to the MCL algorithm and scalability, the authors show in supplementary table S2 that graph construction takes >1 hour in their bench-marking analysis. How does this scale as the size of the input increases (similar to previous reviewers comments)? This could pose a major bottleneck to the adoption of the authors method, particularly compared to the construction of nearest-neighbour graphs which are considerably faster.

**Authors Response**

**Point-by-point responses to the reviewers’ comments:**

Referee 2:

“Whilst the authors validate the presence of the CP population experimentally, the assumption that the other rare cell groups that they identify are equally valid does not hold logically. The authors should demonstrate by one of the above methods that removing probable doublets does not appreciably alter their rare-cell population identification”.

*Although interesting, this suggestion would require a major computational effort from our side, which would delay significantly the publication of our paper. In addition, this work would not change the main conclusions of our paper on the benchmarking and simulated data and very likely only result in minor changes for the neuronal dataset section (if at all). Please see the scientific points that listed at the bottom of this email for more details.*

*At this stage of the publication procedure, and with the two papers on doublet detection methods mentioned by referee 2 published back to back last month only (April 2019), we propose that this request is beyond the scope of our publication. The field is evolving at extremely fast pace and there will always be novel methods we could try to incorporate.*

*To address referee 2’s comment, we instead followed the recommendation by reviewer 3 on doublet (please see below) and include these points in the discussion section of our manuscript”:*

*“Single cell RNA-seq data usually contains a small fraction of doublets, i.e. transcriptomes derived from two or more cells, which could form artifactual clusters. Our results do not indicate the presence of doublet-driven clusters – each subcluster has its own unique markers. In addition, most of the subpopulation signatures represent biological function that are supported by the literature. Finally, we experimentally validated the presence of CP neuroepithelia in our 3D cortical spheroid cultures by confocal microscopy and validated the CP-specific signature gene list identified by CellSIUS using primary pre-natal human data.”*

*The additional work on doublets will not change the main conclusions of our paper and likely only result in minor changes for the NS section based on the following points:*

*• We refer to the minor comment of reviewer 3: "If single cell RNA-seq data usually contain a small fraction of doublets, i.e. transcriptomes derived from two or more cells, which can form artifactual clusters. I don’t think this is a concern in their case, because Fig. S6A does not indicate the presence of doublet-driven clusters – each subcluster has its own unique markers. Nevertheless, they should address this point in the text”.*

*• Of the 7 sub-populations identified by CellSIUS in the NS data, 1 subpopulation has been validated experimentally (G.sub_1) and most of the other subpopulation signatures (including G.sub_3, NP.sub, N.sub_1 and N.sub_2) represent biological function that are supported by the literature and described in the text. In addition, many of those population sizes is above a realistic projection of the content of doublet that could be present in our data.*

*• The focus of our paper is on the development and benchmarking of a novel, sensitive and specific approach for rare cell type identification and characterization. Because of the lack of a ground truth in the NS dataset, we adapted the comparison to other approaches (RaceID3 and Giniclust2) to rare cell population which we had validated experimentally or those whit signatures known from literature (as described in the text).*

*• Our single cell RNAseq data QC involved stringently filtering on detected number of transcripts and genes, that will reduce the number of doublets cells that are outliers for these metrics.*

“With respect to the MCL algorithm and scalability, the authors show in supplementary table S2 that graph construction takes >1 hour in their bench-marking analysis. How does this scale as the size of the input increases (similar to previous reviewers comments)? This could pose a major bottleneck to the adoption of the authors method, particularly compared to the construction of nearest-neighbor graphs which are considerably faster.”

*Table S2 summarizes the running times of several clustering algorithms applied to scRNAseq data, as run with the specifications mentioned below Table S2. Most importantly, these algorithms were run on the entire dataset without any modification or optimization to the packages that are readily available for R. In contrast, the input for CellSIUS is a pre-clustered dataset, therefore MCL is never run on the entire dataset but only within each cluster.*

*On the scalability on MCL itself: according to the author an optimized implementation has complexity O(Nk^2^) with N the number of nodes and k the number of resources allocated per node (see* [*https://micans.org/mcl/*](https://micans.org/mcl/)*). Nearest neighbor trees have an average complexity of O(logN) for randomly distributed points, approximate nearest neighbor implementations scale even better. However, specific implementations of algorithms with their optimizations can influence the running times for the better or worse, as does available memory. Even though the computational complexity of nearest neighbor algorithms can be better than that of MCL, efficient implementations of both are easily applied to the data at hand and, as mentioned before, for CellSIUS we are only interested in within-cluster comparisons where N is expected to be on the order of several hundred at most.*

*Graph construction based on a correct correlation matrix (as opposed to an estimate of the distance from approximate nearest neighbor algorithms) is most easily done from the full correlation matrix. A quick comparison shows that the calculation of a full columns-wise correlation matrix of 1000 by 50000 random numbers takes on the order of 75 minutes in R and 5.7 minutes in python with numpy, taking the most straightforward approach to achieve the task without any optimization (cor for R, numpy.corrcoef in python). Disregarding any potential IO issues, graph construction (ie, thresholding the matrix and writing out an edge list) itself should take a fraction of the time required for the calculations.*

*In summary, “graph construction” as mentioned in Table S2 needs to be considered (1) for the full data set (which is not the case for CellSIUS), and (2) has at least two different steps that could potentially be further optimized but this is not the scope of this manuscript.*

*We performed an assessment of the scalability of CellSIUS itself, including all the steps involved in the actual implementation. We applied CellSIUS to data sets of increasing numbers of cells from 1000 to 7500 cells, composed of 2 to 15 clusters with 500 cells per cluster. For a system of ~1000–1500 cells (ie, 2 or 3 clusters) the CPU time is of ~10–20 seconds, see plot below.*

*In conclusion, CellSIUS scales well for the systems that it is designed to work with, and the MCL part, including graph generation, does not represent any bottlenecks.*
